# Enhancement of Vivid-based photo-activatable Gal4 transcription factor in mammalian cells

**DOI:** 10.1247/csf.22074

**Published:** 2022-12-16

**Authors:** Shinji C. Nagasaki, Tomonori D. Fukuda, Mayumi Yamada, Yusuke III Suzuki, Ryo Kakutani, Adam T. Guy, Itaru Imayoshi

**Affiliations:** 1 Laboratory of Brain Development and Regeneration, Graduate School of Biostudies, Kyoto University, Kyoto 606-8501, Japan; 2 Research Center for Dynamic Living Systems, Graduate School of Biostudies, Kyoto University, Kyoto 606-8501, Japan; 3 Laboratory of Cell Biology, Institute for Life and Medical Sciences, Kyoto University, Kyoto 606-8507, Japan; 4 Laboratory of Science Communication, Graduate School of Biostudies, Kyoto University, Kyoto 606-8501, Japan; 5 Laboratory of Deconstruction of Stem Cells, Institute for Life and Medical Sciences, Kyoto University, Kyoto 606-8507, Japan

**Keywords:** optogenetics, Gal4/UAS system, transcription, gene expression, Vivid

## Abstract

The Gal4/UAS system is a versatile tool to manipulate exogenous gene expression of cells spatially and temporally in many model organisms. Many variations of light-controllable Gal4/UAS system are now available, following the development of photo-activatable (PA) molecular switches and integration of these tools. However, many PA-Gal4 transcription factors have undesired background transcription activities even in dark conditions, and this severely attenuates reliable light-controlled gene expression. Therefore, it is important to develop reliable PA-Gal4 transcription factors with robust light-induced gene expression and limited background activity. By optimization of synthetic PA-Gal4 transcription factors, we have validated configurations of Gal4 DNA biding domain, transcription activation domain and blue light-dependent dimer formation molecule Vivid (VVD), and applied types of transcription activation domains to develop a new PA-Gal4 transcription factor we have named eGAV (enhanced Gal4-VVD transcription factor). Background activity of eGAV in dark conditions was significantly lower than that of hGAVPO, a commonly used PA-Gal4 transcription factor, and maximum light-induced gene expression levels were also improved. Light-controlled gene expression was verified in cultured HEK293T cells with plasmid-transient transfections, and in mouse EpH4 cells with lentivirus vector-mediated transduction. Furthermore, light-controlled eGAV-mediated transcription was confirmed in transfected neural stem cells and progenitors in developing and adult mouse brain and chick spinal cord, and in adult mouse hepatocytes, demonstrating that eGAV can be applied to a wide range of experimental systems and model organisms.

## Introduction

Gene expression during development, homeostatic maintenance and environmental responses in living cells is highly dynamic. To understand the functional significance of dynamic gene expression changes, light-controllable gene expression systems have been developed and are being continuously updated (Crefcoeur *et al.*, 2013; Hallett *et al.*, 2016; Hörner *et al.*, 2017; Konermann *et al.*, 2013; Motta-Mena *et al.*, 2014; Pathak *et al.*, 2017; Polstein and Gersbach, 2012; Shimizu-Sato *et al.*, 2002; Wang *et al.*, 2012; Yazawa *et al.*, 2009; Quejada *et al.*, 2017; Yamada *et al.*, 2018; Liu *et al.*, 2012; Müller *et al.*, 2013b; Zhou *et al.*, 2022; Yamada *et al.*, 2020; Kasatkina *et al.*, 2022; Kuwasaki *et al.*, 2022). Light-controlled gene expression systems are versatile tools to manipulate cellular functions at fine spatiotemporal resolution (Imayoshi *et al.*, 2013; Chan *et al.*, 2015; Nihongaki *et al.*, 2017; Isomura *et al.*, 2017; Shao *et al.*, 2018; Yoshioka-Kobayashi *et al.*, 2020; Zhou *et al.*, 2022).

The Gal4/UAS system is one of the most versatile candidates for light-controllable gene expression system, because it has been commonly applied to fly, zebrafish and mammalian model organisms (Fischer *et al.*, 1988; Brand and Perrimon, 1993). The Gal4/UAS system is a binary gene expression system consisting of Gal4 transcription activator and its target upstream activation sequence (UAS). Gal4 has a DNA-binding domain (DBD) and a transcription activation domain (AD), and specifically binds to the UAS sequence to activate transcription from a basal promoter placed downstream of UAS. This exogenous gene expression control system has been widely used to induce a high level of expression of a gene of interest in a specific cell type with temporal control.

Recently, various types of photo-activatable (PA) molecules such as Cry2-CIB1 (Kennedy *et al.*, 2010; Aoki *et al.*, 2017; Taslimi *et al.*, 2016; Quejada *et al.*, 2017), Vivid (VVD) (Wang *et al.*, 2012), Magnet (Kawano *et al.*, 2015; di Pietro *et al.*, 2021), tunable light-controlled interacting protein tags (TULIPs) (Strickland *et al.*, 2012), and original light-inducible dimer/improved light-inducible dimer (oLID/iLID) (Guntas *et al.*, 2015; Hallett *et al.*, 2016) have been incorporated to the Gal4/UAS system to develop blue-light inducible PA-gene expression systems. In addition, optical switches responsive to other wavelengths of light such as ultraviolet (Müller *et al.*, 2013a) or near-infrared (Kaberniuk *et al.*, 2016; Redchuk *et al.*, 2017; Kasatkina *et al.*, 2022; Kuwasaki *et al.*, 2022) light, have been integrated for multi-wavelength controls of gene expression systems including the Gal4/UAS system.

In this study, we designed blue light-controllable Gal4 transcription activator molecules whose active dimer formation process is mediated by the *Neurospora crassa* photoreceptor VVD. VVD is the smallest light-oxygen-voltage (LOV) domain-containing protein, and generates a rapidly exchanging homodimer under blue-light activation (Zoltowski *et al.*, 2007). By comprehensive functional screening of candidate constructs, we have identified the optimized PA-Gal4 transcription activator for achieving the precise manipulation of gene expression at fine spatiotemporal resolution in mammalian and avian cells.

## Materials and Methods

### Constructs

For functional screening of eGAV candidate constructs, the sequences encoding DNA-binding domain (DBD) of Gal4 (residues 1–65) were amplified from pEF-hGAVPO (Imayoshi *et al.*, 2013; Yamada *et al.*, 2020; Wang *et al.*, 2012) using polymerase chain reaction (PCR) method. The Vivid (VVD) photodimerization domain (residues 37–186) with mutations (N56K and C71V) were also amplified from pEF-hGAVPO. In the validation of transcription activation domains (ADs), the DNA sequences encoding p65 (residues 286–550 of human p65), VP16 (residues 413–490 of herpes simplex virus transcription factor VP16) and VP64 (tandem 4-copy repeats of VP16 AD), Rta (replication and transcription activator of human gammaherpesvirus 4), HSF1 (human heat shock transcription factor 1), VPR (a tripartite activator VP64-p65-Rta), VPRmini (VPR miniature activator), were applied (van Essen *et al.*, 2009; Sadowski *et al.*, 1988; Seipel *et al.*, 1992; Noda and Ozawa, 2018; Beerli *et al.*, 1998; Regier *et al.*, 1993; Lu *et al.*, 2006; Barna *et al.*, 2018; Kunii *et al.*, 2018; Chavez *et al.*, 2015; Vora *et al.*, 2018). The coding sequences of VP16 and VP64 were gifted from Dr. Takeaki Ozawa (The University of Tokyo) (Noda and Ozawa, 2018). The coding sequence of p65 and Rta was amplified from pEF-hGAVPO and PB-TRE-dCas9-VPR plasmid, respectively. PB-TRE-dCas9-VPR was a gift from Dr. George Church (Wyss Institute) (Addgene plasmid #63800) (Chavez *et al.*, 2015). The coding sequence of HSF1 was gifted from Dr. Tetsushi Sakuma (The University of Hiroshima) (Kunii *et al.*, 2018). For VPR (Chavez *et al.*, 2015), the coding sequence of VP64, p65, and Rta were amplified using PCR method and the three fragments fused in an assembly reaction using NEBuilder HiFi DNA Assembly (E2621, New England Biolabs, Inc., Ipswich, USA). For VPRmini (Vora *et al.*, 2018), the coding sequences of VP64 and p65 (residues 100–261) and Rta (residues 125–190) were amplified using PCR method, and the three fragments were fused in an assembly reaction. Using these sequences, the Gal4 DBD and transcription AD were fused to VVD, and these constructs were cloned into expression vector plasmids with the human elongation factor 1α (EF) promoter sequence and polyadenylation sequences (pEF-BOS) and their derivatives (Mizushima and Nagata, 1990). The three tandem flexible Glycine–Serine (3x GS) linker or restriction enzyme (RE) target sites were inserted between each protein domain. All prepared constructs were verified by DNA sequencing. The enhanced thermostable variants of Magnet (eMags) were codon-optimized for human (Fasmac, Kanagawa, Japan) and eGAV constructs using eMags were generated by the same procedures.

In the plasmid constructions for lentivirus vectors, the EF promoter sequence of the CSII-EF-MCS-IRES2-mCherry-Nuclear Localization Sequence (NLS) plasmid (Yamada *et al.*, 2018; Imayoshi *et al.*, 2013; Miyoshi, 2004) was replaced with the CAG promoter sequence amplified using PCR method from CSII-CAG-MCS (Yamada *et al.*, 2018; Imayoshi *et al.*, 2013; Miyoshi, 2004). The eGAV coding sequence was inserted into multiple cloning sites of this CSII-CAG-MCS-IRES2-mCherry-NLS plasmid. For the UAS reporter lentivirus constructs, CSII-EF-MCS (Miyoshi, 2004) was digested with AgeI to remove the EF promoter sequence, and the 5x UAS sequence and the 3' untranslated region (UTR) of the mouse *Hes1* gene were cloned in the opposite orientation to long terminal repeat (LTR)-mediated transcription. The Ub-NLS-Luc2 coding sequence was inserted immediately after the 5x UAS sequence. The cHS4 insulator-pEF-Puro-Halo sequence was inserted upstream of the 5x UAS sequence for puromycin and HaloTag^®^ (Promega, Madison, USA, G6050) selection. For the fluorescent reporter lentivirus vector construction for the adult mouse brain experiment, CSII-5x UAS-Ub-NLS-Luc2-Hes1 3' UTR was digested with PacI and AscI to remove the Ub-NLS-Luc2 cording sequence, and human codon-optimized PEST degradation sequence from mouse ornithine decarboxylase (PEST) is applied to Achilles as a destabilizing sequence using PCR method, and the coding sequence of destabilized Achilles (Achilles-NLS-PEST) (Yoshioka-Kobayashi *et al.*, 2020) is inserted. The Achilles coding sequence was kindly gifted from Dr. Atsushi Miyawaki (RIKEN Center for Brain Science).

For the construction of the UAS reporter plasmids used for transient transfection, the 5x UAS-Ub-Luc2-Hes1 3' UTR sequence was amplified by PCR method and inserted into pSP72 vector (Promega, P2191). To construct the 5x UAS-Achilles-NLS-PEST-Hes1 3' UTR plasmid, PB-TRE-dCas9-VPR (Addgene plasmid #63800) was digested with SpeI and ApaI to remove the TRE-dCas9-VPR cording sequence, and the 5x UAS-Achilles-NLS-PEST-Hes1 3' UTR coding sequence was inserted using PCR method.

To construct plasmids for *in ovo* electroporation, the EF promoter sequence of the pEF-eGAV plasmid was replaced by the CAG promoter sequence amplified from the CSII-CAG-MCS plasmid using PCR method. The CAG-eGAV plasmid was digested with SalI and NotI, and the mRuby3 coding sequence was amplified from the mRuby3-C1 plasmid using PCR method to construct CAG-mRuby3. mRuby3-C1 plasmid was a gift from Dr. Salvatore Chiantia (Addgene plasmid #127808) (Dunsing *et al.*, 2018; Bajar, 2016).

### Cell culture

Human embryonic kidney 293T (HEK293T) cells, NIH3T3 mouse embryonic fibroblasts and EpH4 mouse mammary epithelial cells (American Type Culture Collection [ATCC], Manassas, USA) were cultured at 37°C and 5% CO_2_ in Dulbecco’s Modified Eagle’s Medium (DMEM; Nacalai Tesque, Kyoto, Japan 08458-16; ThermoFisher, Waltham, USA, 11039047) supplemented with 10% fetal bovine serum (FBS; ThermoFisher, Hyclone, UT, Logan, SH30071.03; Sigma-Aldrich, St. Louis, USA, S173012, Lot; S. BCCC3916) and 100 units/mL penicillin and 100 mg/mL of streptomycin (Nacalai Tesque, 09367-34). HEK293T and NIH3T3, and EpH4 cells were passaged using 0.05% and 0.25% Trypsin/EDTA (Nacalai Tesque, 32778-05 and 32777-15), respectively.

### Mice

All animal protocols were approved by the Animal Care Committees of the Kyoto University (permit number: Lif-K22008). All animal experiments were performed in accordance with the principles outlined in the Kyoto University Guide for the Care and Use of Laboratory Animals. ICR embryos (Japan SLC, Hamamatsu, Japan) were used for the analysis in the embryonic brain. Male 12-week-old C57BL/6 mice (Japan SLC) were used for the analysis in the adult mouse brain. Female 4-week-old ICR mice (Japan SLC) were used for the analysis in the liver tissue. The mice were group housed in a standard laboratory environment, maintained on a 14 h light/10 h dark cycle at a constant temperature (23–24°C) and relative humidity (40%–50%). Food (pellets; Japan SLC) and water were provided *ad libitum*.

### Lentivirus packaging

Lentiviral particles were produced via transfection of HEK293T cells with packaging plasmids as previously described (Imayoshi *et al.*, 2013; Miyoshi, 2004). Viral titers were approximately 10^8–9^ infectious units/mL. Cultured cells were infected by purified lentiviral particles with a multiplicity of infection (MOI) = 10–50. Transduced EpH4 cells were selected by fluorescence-activated cell sorting (FACS) (BD Biosciences, Franklin Lakes, USA, FACSAriaIIIu) for cells co-expressing mCherry and HaloTag^®^ (Promega) selection. For transduced HEK293T cells, Achilles-positive cells were selected by FACS (BD Biosciences, FACSAriaIIIu) after exposure to blue light (7.10 W m^–2^) for 6 h (30 s on and 180 s off cycles).

### Light source

For blue light irradiation of cultured cells in CO_2_ incubators, we used a 465 nm Light-emitting diode (LED) (Nichia, Anan, Japan, NCSB119) assembly array (Ebisu Electronics, Hirakata, Japan) and an LED blue light source (OptoCode, Ota, Japan, LEDB-SBOXH). For blue light illumination under the microscope, blue light was generated by a pE-2 LED excitation system (CoolLED, Andover, UK) equipped with a 470 nm LED Array Module (LAM). For blue light illumination of the adult mice brain, we use a 470 nm LED (Thorlabs, Newton, USA, M470F3) connected to the fiber optic cannula (Thorlabs, CFMLC52L02) via fiber patch cables and a rotary joint. Light intensity was measured using a light meter (Thorlabs, PM100A, S120VC).

### Luciferase assays

Luciferase activity of the lysed cells was assayed according to the manufacturer’s instructions (Promega, Luciferase Assay System, E1501).

### Live-cell monitoring of luciferase activity

Luminescence signals at the population level were recorded by a live cell monitoring system (Churitsu Electric Corp., Nagoya, Japan, CL24B-LIC/B) equipped with a highly sensitive photomultiplier tube (PMT) and an LED blue light source (OptoCode, LEDB-SBOXH). Cells were plated on black 24-well plates (BM Equipment, Koto, Japan, 303012) in the culture medium containing 1 mM luciferin (Nacalai Tesque, 0149385), and recorded.

### Luciferase imaging

Cells or organotypic slices were incubated on 35-mm glass bottomed dishes (AGC Techno Glass, Haibara, Japan, 3910-035) at 37°C in 5% CO_2_. 1 mM luciferin was then added to the culture medium. Bioluminescence images were acquired on an upright microscope (Evident, Shinjuku, Japan, IX83) with a 10× or 40× dipping objective. Digital images were acquired using a cooled Charge-Coupled Device (CCD) camera (Andor, Belfast, UK, iKon-M DU934P-BV). The filters and camera control were adjusted automatically using software (Universal Imaging Corp., Bedford Hills, USA, MetaMorph^®^). Stray light was eliminated by turning off the electric system. The imaging system was used in a dark room.

### Characterization of eGAV

For functional screening of the eGAV candidate constructs, HEK293T cells were plated at 6 × 10^4^ cells/well or NIH3T3 cells were plated at 2.5 × 10^4^ cells/well in a 24-well plate. The cells were cultured for 24 h at 37°C in 5% CO_2_ and then transfected with polyethylenimine (Polysciences, Inc., Warrington, USA) according to the manufacturer’s instructions. Two plasmids were co-transfected at a 4:1 ratio: pEF-eGAV candidates, and 5x UAS-Ub-NLS-Luc2-Hes1 3' UTR reporter (Imayoshi *et al.*, 2013). Expression plasmids of Gal4 DBD without any AD and pBluescript plasmid were used for negative control experiments. The total amount of DNA was 0.5 μg/well. Twenty-four hours after transfection, the cells were exposed to blue light (7.10 W m^–2^) for 24 h (30 s on and 180 s off cycles). Thereafter, cells were lysed and their luciferase activity was measured using a plate reader (Promega, GloMax Explorer). Control cells were kept in the dark after plasmid transfection. The pEF-Gal4-VN8x6 plasmid was used as a light-independent Gal4 transcription activator (Salghetti *et al.*, 2000).

To analyze the relationship between the irradiated blue light power and the level of induced gene expression, HEK293T cells were plated in a 24-well plate at 6 × 10^4^ cells/well and transfected. Twenty-four hours after transfection, blue light was applied to cells for 24 h. Light irradiation conditions were cycles of 30 s on and 180 s off at a light intensity of 0.71 or 1.40 or 7.10 W m^–2^. Twenty-four hours after the start of light irradiation, cells were lysed and their luciferase activity measured.

To establish the temporal characteristics of eGAV, transfected HEK293T cells were used. Cells were plated in black 24-well plates (BM Equipment, 303012) and exposed to blue light (7.10 W m^–2^) for 2 min. Luminescence signals at the population level were recorded by a live-cell monitoring system (Churitsu Electric Corp., CL24B-LIC/B). For monitoring transiently transfected cells, HEK293T cells were plated at 1 × 10^4^ cells/well and transfected 24 h later. The first blue light illumination was initiated 36 h after transfection.

For the validation of the eGAV system in the neural stem cells and progenitors of the developing mouse brain, the pEF-mCherry-NLS, pEF-eGAV and 5x UAS-Ub-NLS-Luc2-Hes1 3' UTR reporter plasmids were mixed at a 2:9:9 ratio, and co-transfected into dorsal telencephalic progenitors by *ex utero* electroporation (Imayoshi *et al.*, 2013) at embryonic day 14. The plasmid mixture (2.5 μg/μl) was microinjected into the telencephalic ventricle, and *ex utero* electroporation (6 pulses, 50 V) was performed using square wave generator (Nepagene, Ichikawa, Japan, NEPA21) and 5-mm paddle electrodes for transfection of plasmids into neural stem cells and progenitors at the ventricular surface of the neocortex. Brains were immediately dissected, embedded in 3% low-melting point agarose, cut into 250-μm organotypic slices with a vibratome (Leica, Wetzlar, DE, VT1000), transferred to 12-mm well culture insert (Merck, Darmstadt, DE, PICM01250), and cultured in slice culture medium, DMEM/F-12 (GIBCO, Waltham, USA, 11039) supplemented with 0.6 mmol/L L-Glutamine, 5% horse serum, and penicillin/streptomycin. Slices were incubated at 37°C, 5% CO_2_ for 24 h, and then subjected to the light illumination experiments.

### For hydrodynamic transfection of the liver

Female 4-week-old ICR mice (Japan SLC) were used for the experiments. 2 mL of phosphate-buffered saline (PBS) containing plasmid DNA (10 μg of pEF-eGAV or pEF-hGAVPO, 30 μg of 5x UAS-Achilles-NLS-PEST-Hes1 3' UTR reporter and 10 μg of pEF-mCherry-NLS) were injected through the tail vein, as previously described (Liu *et al.*, 1999). In the light-irradiated group, the abdominal fur of the mice was removed 2 h after transfection. The mice were then noninvasively light irradiated with 465 nm LED (Nichia, NCSB119) assembly array (Ebisu Electronics) for 20 h (1 min on and 1 min off cycles). Light intensity was 90 mW cm^–2^ as previously reported (Wang *et al.*, 2012). Mice were kept in free-moving conditions. Fluorescence images of mCherry and Achilles were acquired with a macro zoom microscope (Evident, MVX10).

### Mouse brain study

For the validation of the ability of eGAV in neural stem cells and progenitors of the adult brain, mice were subjected to stereotactic virus injections, as described previously (Yamada *et al.*, 2020; Kawashima *et al.*, 2013; Sano and Yokoi, 2007). Twelve-week-old C57BL/6 male mice (Japan SLC) were anesthetized with a cocktail of 0.3 mg/kg medetomidine (Nippon Zenyaku Kogyo, Koriyama, Japan), 4.0 mg/kg midazolam (Sandoz, Minato, Japan), and 5.0 mg/kg butorphanol (Meiji Seika Pharma, Chuo, Japan) by intraperitoneal injection. The stereotactic injections were administered to the following tissue at the appropriate coordinates: the dentate gyrus of the hippocampus (A/P –2.0 mm, M/L ±1.3 mm from the bregma, D/V –1.80 mm from the pial surface). The two lentivirus vectors were co-transduced at a 1:1 ratio: CSII-CAG-eGAV-IRES2-mCherry-NLS-WPRE and CSII-5x UAS-Achilles-NLS-PEST-Hes1 3' UTR. After the injection, the skin incision was sutured and treated with antibiotic cream, and an analgesic was injected subcutaneously to relieve post-surgical pain. The post-injection animals were bred normally for 7 days before blue light exposure. Light stimulation was started 7 days after lentivirus transduction. For the hippocampus-light illumination of the adult mice, awake mice were stimulated using a blue LED (Thorlabs, Newton, USA, M470F3) connected to the fiber optic cannula (Thorlabs, CFMLC52L02) via fiber patch cables and a rotary joint at the appropriate coordinates (A/P –2.0 mm, M/L ±1.3 mm from the bregma, D/V –1.20 mm from the pial surface). Light intensity was measured using a light meter (Thorlabs, PM100A, S120VC) and light irradiation was performed for 12 h at 12.6 μW (30 s on and 180 s off cycles). After the blue light exposure, mice were transcardially perfused with 4% paraformaldehyde/PBS. The dissected brains were subjected to immunohistochemistry.

### Immunofluorescence staining

Brain tissues were fixed with 4% paraformaldehyde/PBS overnight at 4°C and then incubated for 48 h at 4°C in 30% sucrose, embedded in OCT (Sakura finetek Japan, Chuo, Japan, Tissue TEK), and frozen. The frozen brains were then sectioned to 50 μm and subjected to immunofluorescent staining. Brain sections were washed with PBS, then blocked and permeabilized with 5% normal donkey serum (NDS) and 0.3% Triton X-100/PBS at room temperature for 1 h, incubated with the primary antibody (rabbit polyclonal anti-GFP, Invitrogen, Waltham, USA, A11122) diluted in 0.3% Triton X-100/PBS containing 1% NDS overnight at 4°C, washed with PBS, and then incubated with regular secondary antibodies conjugated to Alexa 488 (Invitrogen) for 2 h at room temperature. Stained sections were photographed with a confocal microscope (Zeiss, Oberkochen, DE, LSM880).

### *In ovo* electroporation

Animal handling and experimental protocols were approved by the Animal Care Committee of Kyoto University (permit number: Lif-K22021). Fertilized Boris Brown eggs were obtained from a local supplier (Nihon Layer, Gifu, Japan) and maintained in a humidified rocking incubator at 37°C for 3 days until they reached Hamburger and Hamilton Stage 18 or 19 (Hamburger and Hamilton, 1951). *In ovo* electroporation was carried out essentially as previously described (Baeriswyl *et al.*, 2008). Eggs were windowed and the central canal of the embryonic spinal cord was microinjected at the level of the hindlimbs with a plasmid mixture of CAG-eGAV (0.5 μg/μl), 5x UAS-Achilles-NLS-PEST-Hes1 3' UTR reporter (1.0 μg/μl) and CAG-mRuby3 (0.5 μg/μl) in PBS with 0.01% Fast Green dye. Injected spinal cord was transfected by electroporation (5 pulses, 26V) using a NEPA21 square wave pulse generator (Nepagene). Following transfection, the egg shell window was sealed with translucent tape and embryos were incubated in the dark at 37°C. Twenty-four hours after transfection, embryos were irradiated for 24 h with blue light intensity of 42.0 W m^–2^ using 465 nm LED (Nichia, NCSB119) assembly array (Ebisu Electronics) using the 30 s on and 180 s off cycles. Fouty-eight hours after transfection, embryos were screened for plasmid expression and imaged by a macro zoom microscope (Evident, MVX10) and a confocal microscope (Zeiss, LSM880).

### Flow cytometry analysis

To evaluate fluorescent reporter activity, transduced HEK293T cells were irradiated with the blue light pulses (7.10 W m^–2^) for 2 min with 3 h period, and collected at the time of initial light exposure and every 30 min thereafter for 12 h. Cells were trypsinized and collected, then fixed with 3.2% paraformaldehyde/PBS for 15 min at room temperature. The signal intensity of Achilles was then measured using a flow cytometer (SONY, Minato, Japan, Cell Sorter MA900). Fixed HEK293T cells were excited with a 488 nm laser and the signal of Achilles was measured through a 525/50 nm bandpass filter. Based on FSC/SSC parameters, 10000 cells per each sample were analyzed and the means of Achilles signal intensity were calculated. The background was measured using HEK293T cells not infected with the 5x UAS-Achilles-NLS-PEST-Hes1 3' UTR reporter, and the signal intensity of Achilles was calculated by subtracting this background value from each sample data.

### Imaging for fixed cells

HEK293T cells were fixed in 4% paraformaldehyde/PBS for 15 min at room temperature and then washed with PBS. Fluorescence images of Achilles and mCherry were acquired with a confocal microscope (Zeiss, LSM880).

### Image analysis and quantification

Image analysis was performed using ImageJ software and custom plug-ins, as described previously (Yamada *et al.*, 2018; Imayoshi *et al.*, 2013; Isomura *et al.*, 2017).

### Estimation of the activation and deactivation kinetics of light-induced gene expression

The half-lives of the switch-on/off kinetics of light-induced gene expression in eGAV transformed cells were determined as previously described (Yamada *et al.*, 2020). All programs for this analysis were written in MATLAB R2021a (MathWorks Inc., MA, USA).

### Statistical analysis

Statistical analyses were performed with Prism^®^ 8.0 software (GraphPad Software, San Diego, USA). Student’s *t*-test and one-way ANOVA were used for statistical analysis. *P* values less than 0.05 were considered significant. Statistical methods used in the analysis are described in the figure or table legends.

## Result

### Functional screening of enhanced Gal4-VVD transcription factor, eGAV

Currently, GAVPO (Wang *et al.*, 2012) or its human-codon optimized one hGAVPO (Imayoshi *et al.*, 2013) is a commonly used photoactivatable (PA)-Gal4 transcription factor that can induce high levels of light-induced gene expression. However, GAVPO/hGAVPO has significant levels of leaky background transcription activity in dark, reducing its reliability (Nihongaki *et al.*, 2014; Yamada *et al.*, 2020).

Considering previously reported PA-transcription factors (Nihongaki *et al.*, 2017; Yamada *et al.*, 2018; Yamada *et al.*, 2020), rigorous optimization processes are essential for identifying the improved PA-transcription factors, including the selection of functional domains and their configurations. In this study, we aimed to develop more reliable PA-Gal4 transcription factors with superior light-induced gene expression levels and limited dark leakages compared to GAVPO/hGAVPO.

**Fig. 1:**
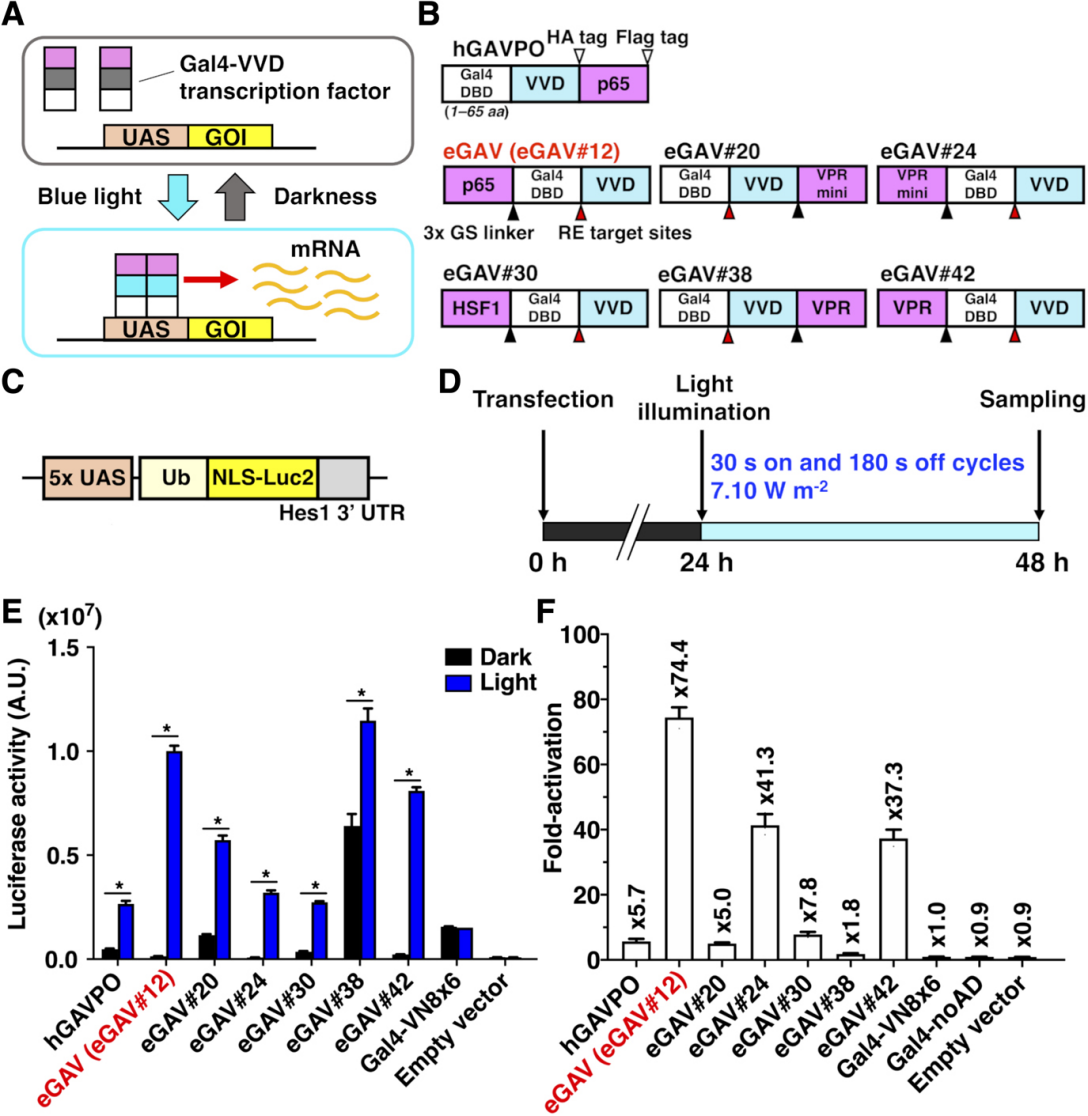
Generation of the enhanced Gal4-VVD transcription factor, eGAV (A) Schematic illustration of blue light-activated transcription of the gene of interest (GOI) triggered by homodimerization of Vivid (VVD). White and magenta rectangles indicate DNA-binding domain (DBD) of Gal4 and transcription activation domain (AD), respectively. Gray and cyan rectangles represent basal and activated state forms of VVD respectively. Gal4-VVD transcription factor binds to its target upstream activation sequence (UAS) by forming homodimers via light-activated VVD and activates transcription from the basal promoter downstream of the UAS sequence. (B) Scheme showing the eGAV constructs. Cyan boxes indicate blue light-dependent dimer formation molecule VVD, and magenta boxes indicate transcription AD, adapted in this study. The DBD of Gal4 (residues 1–65) was used. The three tandem flexible Glycine–Serine (3x GS) linker or restriction enzyme (RE) target sites were inserted between each protein domain. The photoactivatable (PA)-transcription activity of eGAV candidate constructs were compared with human codon-optimized GAVPO (hGAVPO). (C) The reporter construct used in this experiment consisted of 5x UAS, Ub-NLS-Luc2, and Hes1 3' UTR sequences. (D) Experimental time course. (E) Validation of light-dependent regulation of the eGAV constructs in transiently-transfected HEK293T cells. Six candidate constructs which showed prominent light-induced expressions were selected. Functional screening results of all candidate constructs were shown in Fig. S2 and Table S2. The eGAV#12 construct showed relatively high light-induced gene expression and low background activity in the dark, and hereafter simply called as “eGAV”. As negative internal controls, samples transfected with the light non-sensitive Gal4 transcription factor Gal4-VN8x6 (Salghetti *et al.*, 2000), Gal4 DBD without any transcription AD, and empty expression vector were used. The data represent means ± standard deviation (SD) (*n* = 3) from one experiment, and experiments were repeated three times with similar results. Two-tailed Student’s *t*-test was used to compare the reporter activity of the hGAVPO or each eGAV construct in dark and light conditions (*p<0.05). (F) Fold-increase of luciferase activity (Light/Dark). The data represent means ± SD (*n* = 3) from one experiment, and experiments were repeated three times with similar results. See also Fig. S2 and Table S2.

Since the short version of Gal4 DNA binding domain (DBD) (residues 1–65) was reported to be more compatible with PA-Gal4 transcription factors than the long version of Gal4 DBD (residues 1–147) (Yamada *et al.*, 2020), we focused on the sequences containing Gal4 residues 1–65 in the candidate constructs of enhanced Gal4-VVD (eGAV) transcription factor (Fig. 1, S1, Table S1). GAVPO/hGAVPO engages p65 transcription activation domain (AD) derived from human NF-κB (Nuclear factor kappa-light-chain-enhancer of activated B cells) (Wang *et al.*, 2012; Imayoshi *et al.*, 2013). In the optimization of eGAV, we tested additional transcription ADs such as Rta, VP65, VPRmini, HSF1, VP64 and VPR (Chavez *et al.*, 2015; Vora *et al.*, 2018; Kunii *et al.*, 2018; Noda and Ozawa, 2018) (Fig. 1, S1, Table S1). Gal4 DBD (residues 1–65) and transcription ADs were fused to Vivid (VVD), and these constructs were cloned into expression vector plasmids. Also, as for the Flag- and HA-epitope tags inserted in GAVPO/hGAVPO, we did not insert these epitope tags into eGAV because they may alter the function of PA-transcription factors. The three tandem flexible Glycine–Serine (3x GS) linker or restriction enzyme (RE) target sites were inserted between each protein domain. The VVD photodimerization domain (residues 37–186) with mutations (N56K and C71V) which show more efficient light-dependent dimer formation activity (Wang *et al.*, 2012), was used. In the construct screening experiments and functional characterization of the candidate constructs, we applied the destabilized luciferase reporter Ub-NLS-Luc2 (Fig. 1C) and inserted the Hes1 3' untranslated region (UTR) sequence just downstream of Ub-NLS-Luc2. This is known to result in a shorter mRNA half-life and prevent accumulation of the reporter activity in the monitored cells (Luker *et al.*, 2003; Voon *et al.*, 2005; Masamizu *et al.*, 2006).

Of the 42 constructs we tested in human embryonic kidney 293T (HEK293T) cells, 10 constructs showed significant light-dependent increases (>5-fold) of the luciferase transcription reporter (Fig. S2 and Table S2). We selected six constructs for subsequent validation (eGAV-#12, #20, #24, #30, #38, #42 in Fig. 1) because of their low levels of background activity in the dark (#12, #24, #30, #42) or their consistent light-induced gene expression. eGAV-#20 and #38 showed similar as hGAVPO in terms of relatively higher background activity and consistent light-induced gene expression activity. The background activity of eGAV-#24 and #30 was lower than hGAVPO, but the light-induced gene expression levels were moderate. Among the selected constructs, eGAV#12 showed the most prominent light-induced gene expression levels with very limited dark background, and hereafter is simply called as “eGAV”.

We also evaluated these six selected constructs in NIH3T3 mouse embryonic fibroblasts (Fig. S3). Although NIH3T3 cells tended to have lower fold-activation levels than HEK293T cells due to lower transfection efficiency, eGAV#12 showed high light-induced gene expression and low background activity similar to the results in HEK293T cells.

The original VVD makes active homodimers in response to blue light. By amino acid substitutions in VVD, the light-dependent heterodimer formation system Magnet was generated and utilized in various optogenetic tools (Kawano *et al.*, 2015; Benedetti *et al.*, 2020; di Pietro *et al.*, 2021). We changed the VVD sequence of eGAV#12 to eMagnet (eMag), which is an enhanced thermostable variant of Magnet (Fig. S4). However, any pairs of eGAV-eMag showed the extremely high levels of background even in dark conditions, which led to the poor fold-induction values of target gene activity.

### Light power-dependent transcription control of eGAV

**Fig. 2:**
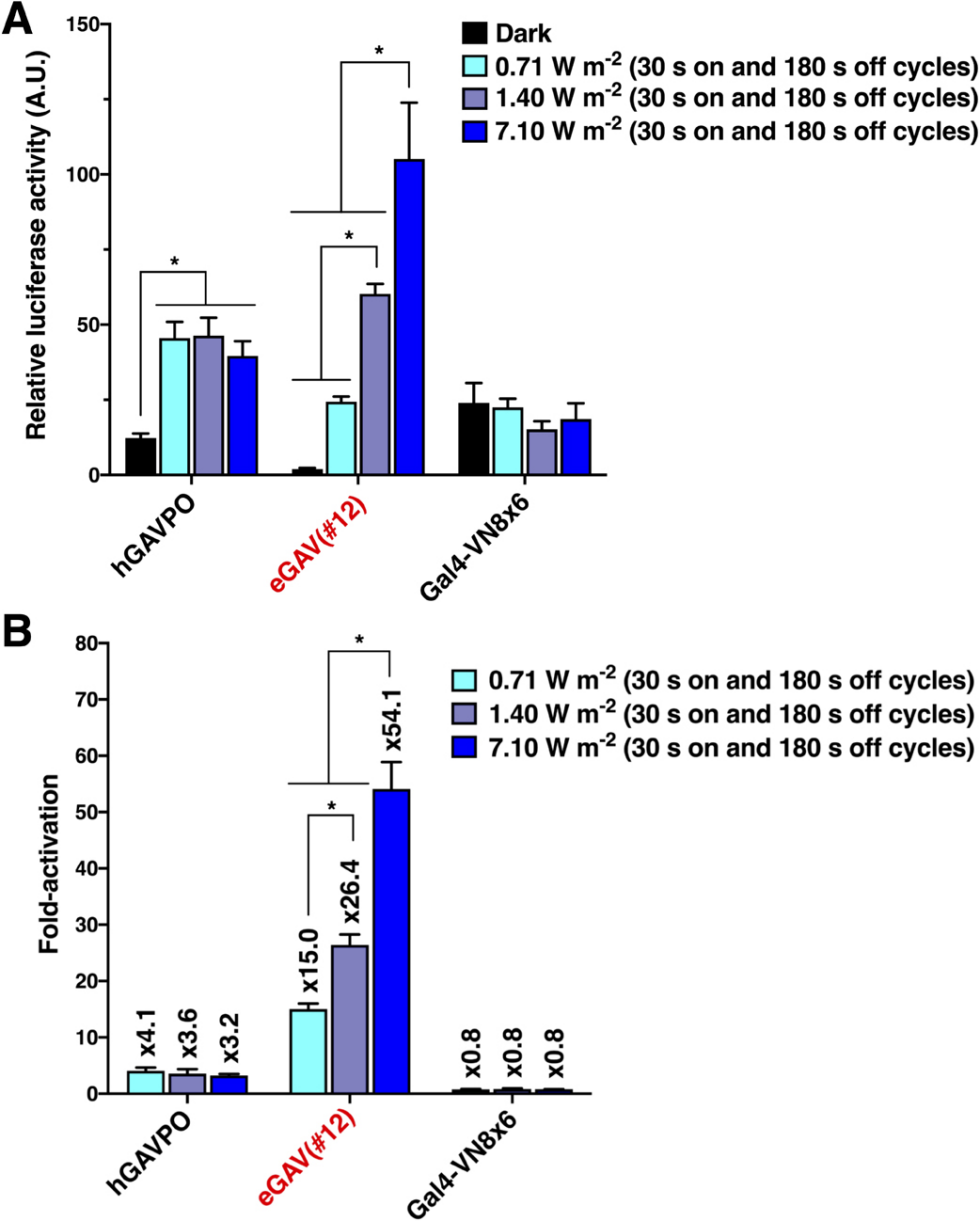
Light dose-dependent regulation of eGAV (A, B) Blue light intensity-dependent transcriptional activity of eGAV in the transiently transfected HEK293T cells. The radiant energy varied from 0.71 to 7.10 W m^–2^. Although the luciferase reporter expression levels were correlated to the illuminated blue light power in eGAV-transfected cells, these light dose-dependent regulations were not observed in hGAVPO-transfected cells. Luminescence values were normalized by that of empty vector-transfected cells in dark conditions. The data represent means ± SD (*n* = 6 for dark and 7.10 W m^–2^, *n* = 3 for all other conditions) from one experiment, and experiments were repeated three times with similar results. *p<0.05; One-way ANOVA followed by Tukey-Kramer’s *post hoc* test.

One major advantage of a photo-activatable gene expression system is the ease of tuning gene expression amount by adjusting light intensity. We investigated the effects of modifying blue light dose on induced-gene expression levels in the eGAV-transfected cells (Fig. 2). We observed an expected blue light intensity-dependent increase of luciferase reporter activity in eGAV-transfected HEK293T cells (Fig. 2), indicating that fine regulation of downstream gene expression was achieved by changing the dose of blue light irradiation. Although light-induced luciferase activities and fold-induction values were increased in correlation with the light intensity in eGAV, such light intensity-dependent regulation was not observed in hGAVPO, consistent with our previous work (Yamada *et al.*, 2020). This indicates more linear light-response properties of eGAV and could offer finer controls of downstream gene expression levels by changing applied blue light power.

### Temporal features of eGAV

**Fig. 3:**
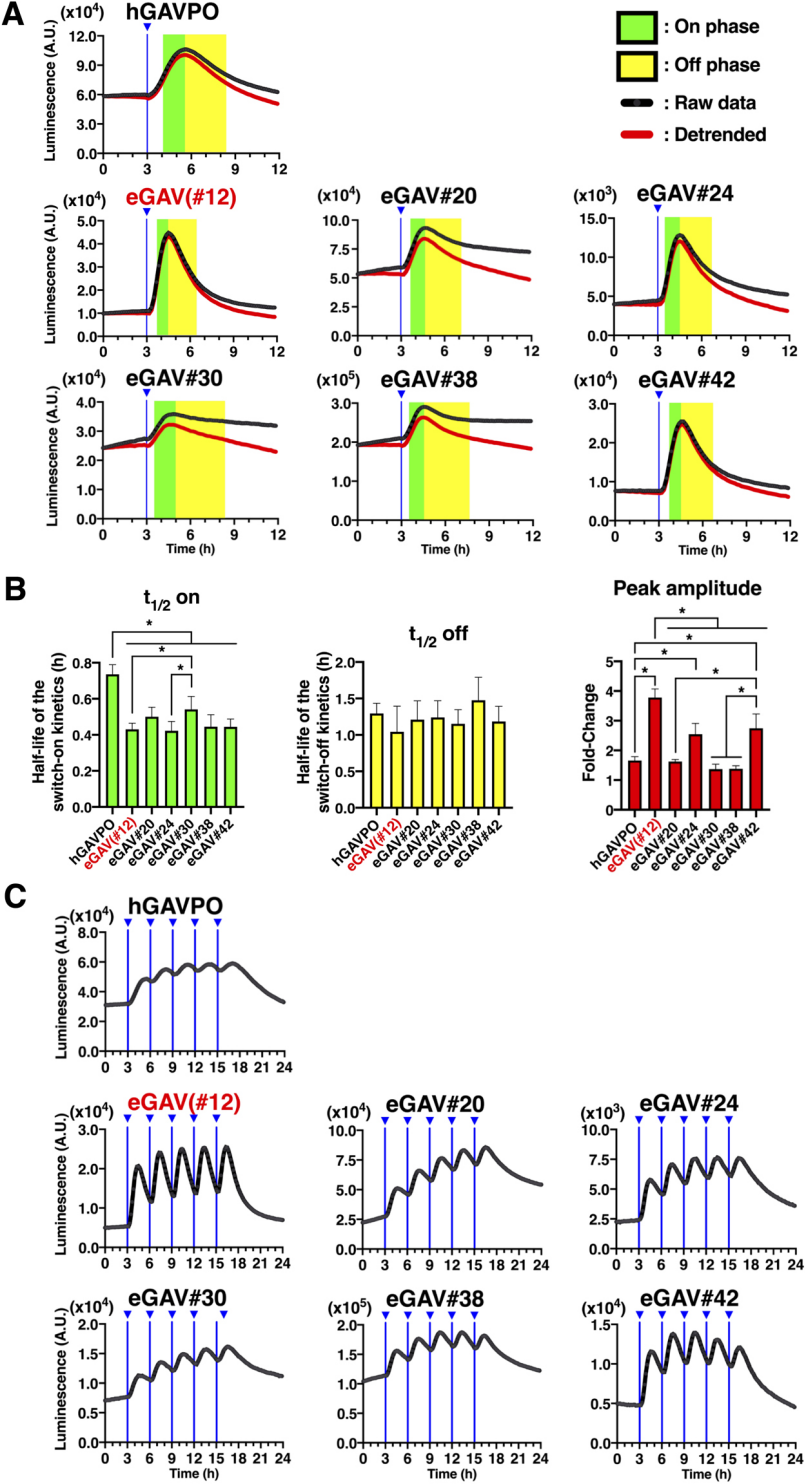
Temporal features of the eGAV transcriptional activators (A) HEK293T cells transfected with the eGAV candidate constructs and 5x UAS-Ub-NLS-Luc2-Hes1 3' UTR reporter were exposed to a single blue light pulse. The blue light was applied to cells 36 h after the transfection. The timing of blue light exposure is indicated by vertical blue lines. The transcription on- and off-phases are highlighted in green and yellow, respectively. The temporal characteristics of eGAV candidate constructs were compared with hGAVPO. (B) Using the single light pulse data set, kymograph analysis was used to determine the half-lives of the switch-on/off kinetics for individual eGAV candidate constructs. For the analysis of amplitude, the baseline value before light irradiation and the maximum peak value after light irradiation were used to determine the fold-change. The data represent means ± SD (*n* = 6). *p<0.05; One-way ANOVA followed by Tukey’s *post hoc* test. (C) Transiently-transfected HEK293T cells, in which eGAV and 5x UAS-Ub-NLS-Luc2-Hes1 3' UTR reporter had been introduced by transiently transfection via lipofection, were repeatedly exposed to blue light pulses at 3 h intervals. The timing of blue light exposure is indicated by vertical blue lines. The first blue light illumination was initiated 36 h after the transfection. One representative experiment is shown, and this experiment was repeated at least three times (*n* = 6).

VVD is rapidly activated to make active homodimer by light illumination, and then spontaneously dissociates with a shorter half-life (Wang *et al.*, 2012). The fast activation and deactivation kinetics of the VVD may allow for dynamic changes of the downstream gene expression of eGAV, such as the periodic oscillatory pattern. We tested the temporal characteristics of eGAV constructs by applying short pulses of light (2 min) and measured the time-course of luciferase expression level, which was under the control of UAS sequences, in real time (Fig. 3). When we analyzed HEK293T cells transiently transfected eGAV#12 and 5x UAS-Ub-NLS-Luc2-Hes1 3' UTR reporter, peak blue light pulse-induced luciferase activity was observed approximately 1.5 h later and returned to background levels 4–5 h later (Fig. 3A, B). Other eGAV constructs also had significantly faster on-kinetics than hGAVPO. Off-kinetics of induced gene expression were not significantly different in individual eGAV constructs. We also evaluated light-induced gene expression activity of eGAV constructs and found that eGAV#12 exhibited the best fold-change values (Fig. 3A, B). Of eGAV constructs, eGAV-#12 and #42 had rapid cycling features and could be suitable to induce complex gene expression dynamics such as oscillatory expression. Indeed, when we periodically applied blue light pulses with 3 h period, robust oscillatory expression was induced in eGAV-#12 and #42 (Fig. 3C). In contrast, eGAV-#20, #24, #30 and #38, which have relatively low light-induced gene expression activity or high background activity, showed a stepwise increase in reporter expression even during the 3 h rapid cycle activation experiment. Considering the smaller molecular size and superior fold-induction of eGAV#12 than eGAV#42, eGAV#12 is the best enhanced VVD-based PA-Gal4 transcription activator.

When we applied different luciferase reporter constructs with longer reporter protein or mRNA degradation half-lives, such as normal Luc2 or simian virus 40 polyadenylation signal (SV40pA), different reporter expression dynamics were induced by the same light illumination protocols (Fig. S5). For example, when we periodically applied blue light pulses with 3 h period to eGAV-transfected cells, robust oscillatory expression was induced with 5x UAS-Ub-NLS-Luc2-Hes1 3' UTR reporter, whereas stepwise increase or sustained types of luciferase activity was observed with 5x UAS-NLS-Luc2-Hes1 3' UTR reporter and 5x UAS-Ub-NLS-Luc2-SV40pA reporter, respectively. Thus, by changing the reporter protein and mRNA half-lives, different gene expression patterns (oscillatory change, step-wise increase, or sustained) can be designed using eGAV.

### Validation of eGAV in tissues and organ

**Fig. 4:**
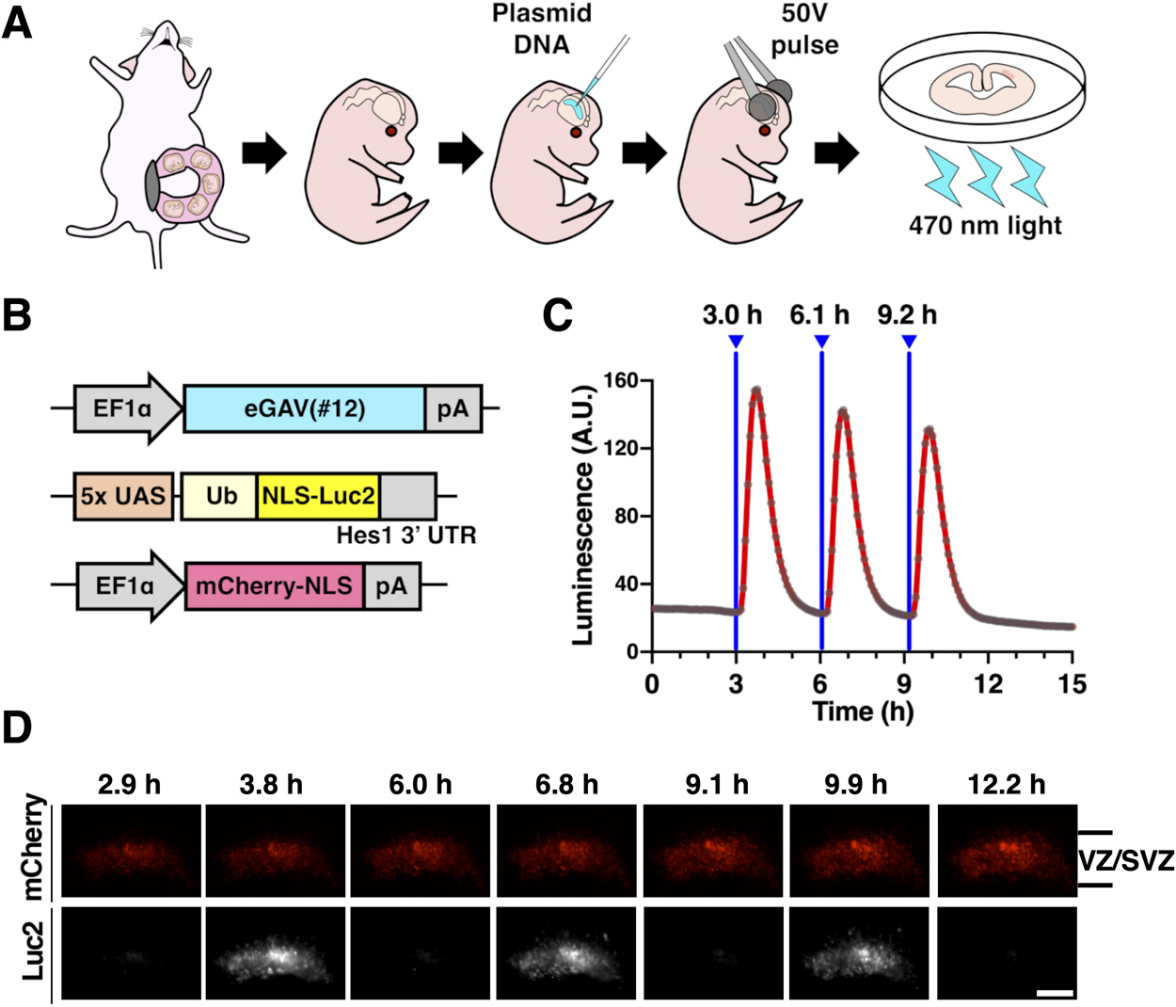
Optogenetic manipulation of gene expression in the organotypic slice culture of embryonic mouse brain by *ex utero* electroporation (A) *Ex utero* electroporation was performed on embryonic mouse brain at embryonic day 14, and the brain was immediately sliced and cultured to evaluate optogenetic manipulation of target gene expression by eGAV. (B) The embryonic mouse brain was transfected with eGAV expression vector, 5x UAS-Ub-NLS-Luc2-Hes1 3' UTR reporter, and mCherry-NLS expression vector as a transfection marker. (C, D) Organotypic brain slices were irradiated with blue light (6.0 μW) for 4 min at 3 h intervals to monitor activity of luminescence reporter. Blue light-induced luciferase expression was observed in neural stem and progenitors in ventricular and subventricular zone. Experiments were repeated three times (*n* = 3). Scale bar, 200 μm.

We then evaluated the ability of the eGAV to light-control gene expression in tissues other than cultured cell lines. To this aim, we tested eGAV activity in neural stem cells and progenitors of the developing mouse forebrain (Fig. 4). We transfected neural stem cells and progenitors with the eGAV expression plasmid and the 5x UAS-Ub-NLS-Luc2-Hes1 3' UTR reporter using *ex utero* electroporation. When brain tissue slice cultures derived from the electroporated brain were periodically irradiated with blue light at 3 h periods, oscillatory reporter activity changes were observed in the ventricular and subventricular zones (VZ/SVZ) where neural stem cells and progenitors preferentially reside (Imayoshi and Kageyama, 2014a, 2014b). These findings suggest that light-manipulation of gene expression using eGAV can manipulate the gene expression pattern of fate determination transcription factors that are oscillatory expressed in neural stem cells and progenitors with high temporal resolution.

**Fig. 5:**
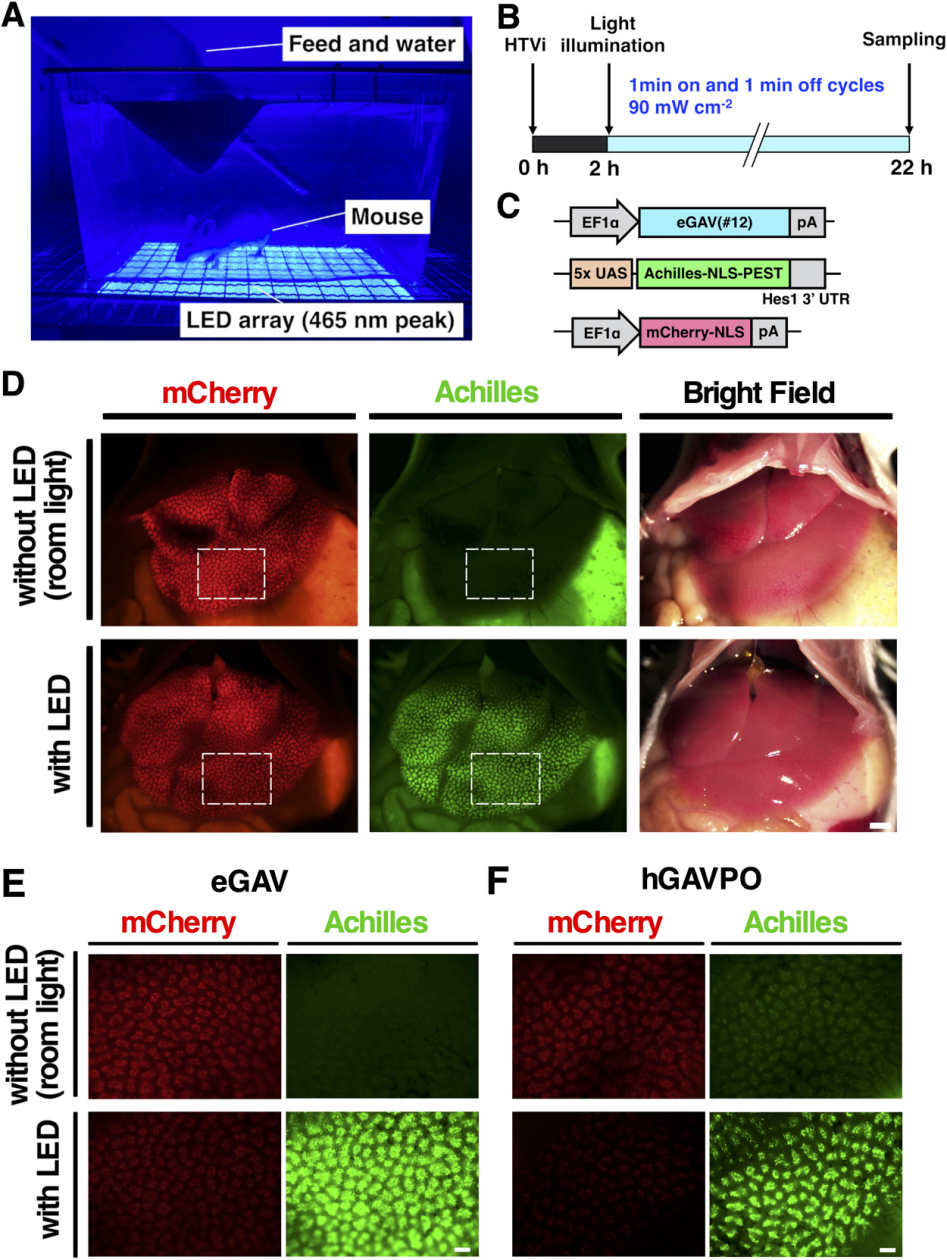
Optogenetic manipulation of gene expression in hepatocytes by HTVi and noninvasive blue light irradiation (A) Photograph of blue light-irradiated mouse after hydrodynamic tail vein injection (HTVi). The abdomen was depilated and irradiated with blue light using an LED array. (B) Time course of the experiment and light irradiation condition. Mice were exposed to blue light for 20 h at 90.0 mW cm^–2^ (1 min on and 1 min off cycles). (C) The mouse hepatocytes were transfected with eGAV expression vector, 5x UAS-Achilles-NLS-PEST-Hes1 3' UTR reporter, and mCherry-NLS expression vector as a transfection marker. (D–F) Representative images of gene expression induced by blue light irradiation in eGAV-transfected mouse liver (D, E) and hGAVPO-transfected mouse liver (F). White boxes in D were enlarged in E. Higher leakage expression of Achilles was observed in hGAVPO-transfected livers than eGAV-transfected livers. Experiments were repeated three times (*n* = 3). Scale bars, 2 mm for (D) and 500 μm for (E, F).

Next, we tested eGAV-mediated light-dependent gene expression in mouse hepatocytes. We transiently transfected mice hepatocytes with the eGAV expression plasmid, the 5x UAS-Achilles-NLS-PEST-Hes1 3' UTR reporter and transfection marker mCherry-NLS expression plasmid using a hydrodynamic tail vein (HTV) injection protocol (Fig. 5). Two hours after HTV injection, transfected mice were irradiated with blue light for 20 h. An increase of the Achilles-fluorescent reporter activity (*green*) was observed in the livers of illuminated mice, but not in mice kept in the dark conditions. Although blue light attenuates as it penetrates the skin, continuous illumination with blue light resulted in a substantial increase in fluorescent reporter activity in the livers of the transfected mice. For leakage activity under the dark conditions, higher fluorescent reporter activity was observed in hGAVPO-transfected livers than in eGAV-transfected livers. We also confirmed robust blue light-dependent gene expression activity of eGAV in embryonic chick spinal cord transfected by electroporation (Fig. S6). After exposure to blue light, the transcription reporter expression was observed in neural progenitors and nascent neurons in the transfected region of spinal cord, but not in embryos in the dark condition.

### Application of eGAV together with lentivirus vectors

**Fig. 6:**
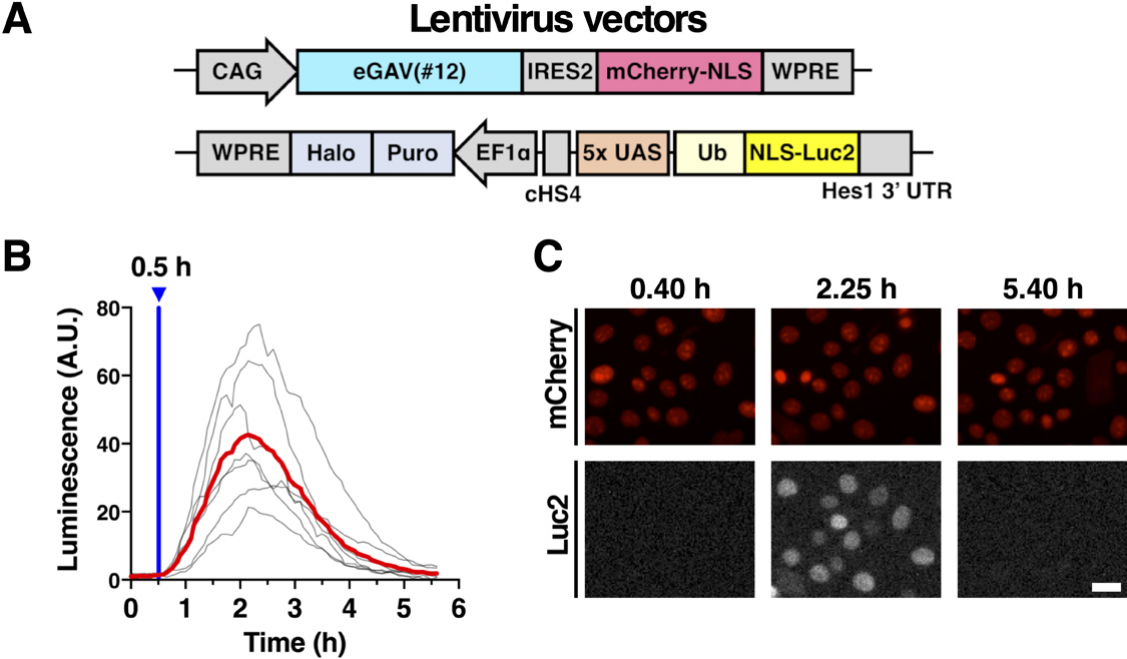
Evaluation of mouse EpH4 cells infected with eGAV-lentivirus vectors (A) The eGAV-expressing mouse EpH4 cells were generated by infection with lentivirus vectors, CAG-eGAV-IRES2-mCherry-NLS-WPRE and 5x UAS-Ub-NLS-Luc2-Hes1 3' UTR reporter. (B) The reporter activity changes in seven cells by blue-light irradiation (6.0 μW) for 4 min were quantified. The gray line is the luminescence value of each cell and the red line shows the mean. Experiments were repeated three times (*n* = 3). (C) Live imaging of reporter expression changes in response to the single blue light pulse. Scale bar, 50 μm.

To enable light-manipulation of target gene expression in the stable eGAV-expressing cells, we used lentivirus vectors to express eGAV and to integrate the reporter construct in EpH4 mouse mammary epithelial cells (Fig. 6). Consistent with the co-transient transfection data of eGAV and destabilized luciferase reporter (Fig. 1, 2, 3), the reporter activity was greatly increased in the stable eGAV-expressing EpH4 cells under blue light illumination (Fig. 6). We also established eGAV-expressing stable HEK293T cells integrated with a destabilized fluorescent reporter, 5x UAS-Achilles-NLS-PEST-Hes1 3' UTR, using lentivirus vectors. Then, when we periodically applied blue light pulses with 3 h period, oscillatory expression of the fluorescent reporter was observed (Fig. S7). These stable eGAV-expressing cells in which destabilized luminescence or fluorescent reporter were integrated with lentivirus vectors showed efficient and reliable blue light-inducible gene expression, and also showed rapid activation/deactivation kinetics (Fig. 6, S7). In addition, similar to hGAVPO (Imayoshi *et al.*, 2013; Yamada *et al.*, 2020; Isomura *et al.*, 2017), one single round of fluorescence-activated cell sorting (FACS) selection was sufficient to generate stable cells expressing eGAV.

**Fig. 7:**
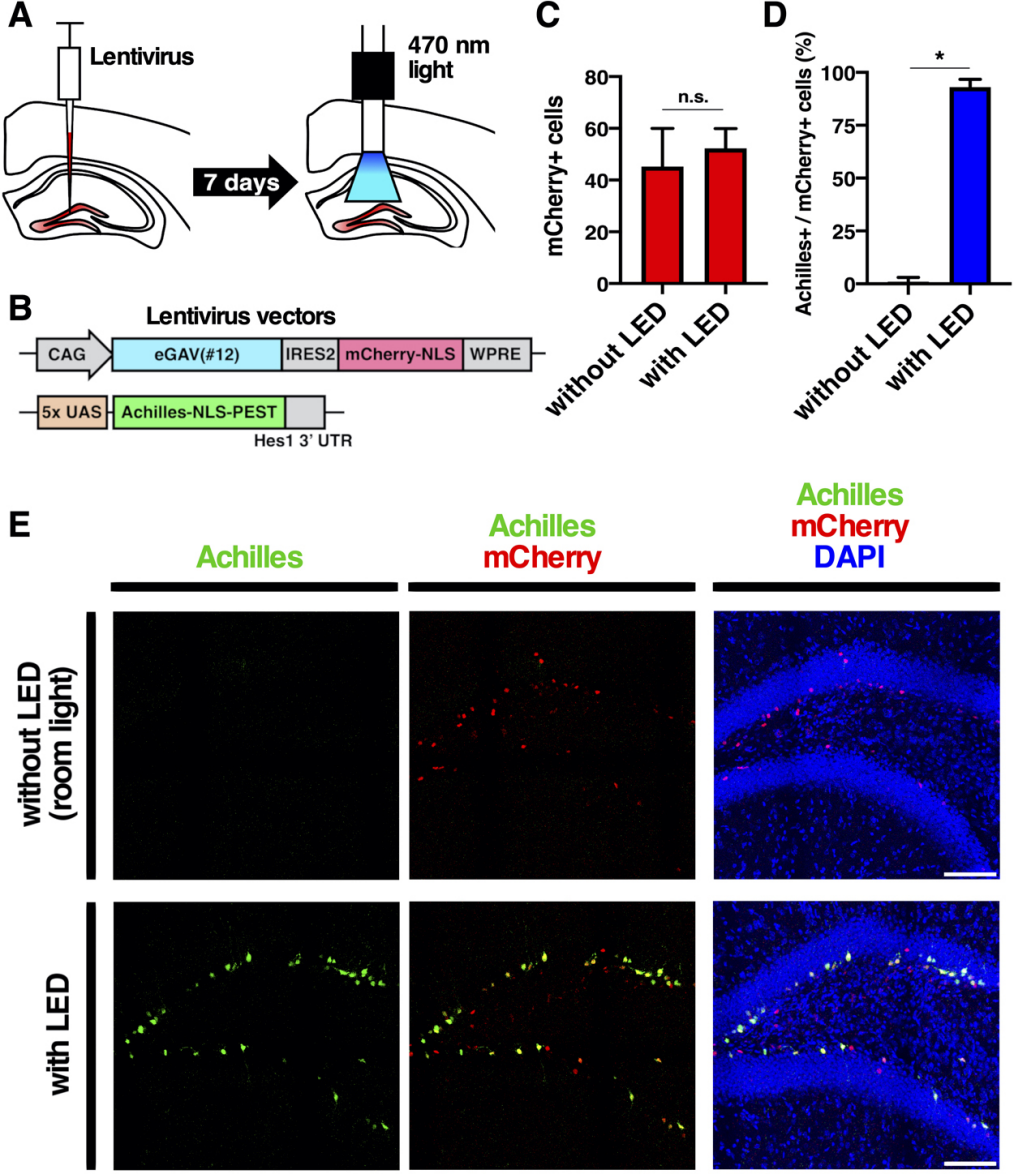
Optogenetic manipulation of gene expression in mouse brain (A) Schematic illustration of the optogenetic experiment using lentivirus vectors. The mouse hippocampal dentate gyrus was irradiated with 470 nm-light (12.6 μW) via optical fiber for 12 h (30 s on and 180 s off cycles). (B) The neural stem cells and progenitors in the mouse hippocampal dentate gyrus were infected with CAG-eGAV-IRES2-mCherry-NLS-WPRE and 5x UAS-Achilles-NLS-PEST-Hes1 3' UTR reporter. (C) The number of cells expressing mCherry (mCherry^+^) in the hippocampal dentate gyrus were counted. The data represent means ± SD (*n* = 4). n.s., not significant; two-tailed Student’s *t*-test. (D) The ratio of Achilles expression in mCherry positive cells was determined. The data represent means ± SD (*n* = 4). *p<0.05; two-tailed Student’s *t*-test. (E) Representative images of blue light-induced Achilles expression in the eGAV-infected mouse dentate gyrus. Scale bars, 100 μm.

Finally, we validated blue light-mediated gene expression control in the intact living mouse brain (Fig. 7). We transduced cells of the hippocampal dente gyrus of adult mouse brain with lentivirus vectors, CAG-eGAV-IRES2-mCherry-NLS-WPRE and 5x UAS-Achilles-NLS-PEST-Hes1 3' UTR reporter. For the blue light activation, hippocampus of awake head-fixed mice was illuminated using a blue LED connected to the optical implant via fiber patch cables and a rotary joint at an intensity of at 12.6 μW for 12 h (30 s on and 180 s off cycles) (Fig. 7). It is known that neural stem cells and progenitors are located at the subgranular zone of the dentate gyrus and that lentivirus vectors preferentially infect these cells (van Hooijdonk *et al.*, 2009; Sueda *et al.*, 2019; Kaise *et al.*, 2022). We found that light-induced gene expression reporter Achilles was expressed in neural stem cells and progenitors in the subgranular zone (Fig. 7E). When not illuminated, neural stem cells and progenitors in the subgranular zone did not express Achilles, indicating successful blue light-induced gene expression by the eGAV PA-transcription factor.

## Discussion

In this study, we describe an enhanced blue light-inducible Gal4 transcription activator eGAV for the spatiotemporal control of gene expression in mammalian cells. We carried out functional screening by investigating the configuration of Gal4 DBD, transcription AD and VVD, and selected final eGAV construct having the enhanced light-induced transcription activity and limited leak activity in dark. When compared to hGAVPO, the most commonly used PA-Gal4 transcription factor, eGAV showed superior maximum induced gene expression levels and significantly lower dark leakage.

To develop eGAV, we used the *Neurospora crassa*-derived blue-light photoreceptor VVD that self-dimerizes through its LOV domain on exposure to blue light. Because VVD is the smallest light-oxygen-voltage domain-containing protein, we speculated that it could be integrated into various designed proteins including synthetic transcription factors. The chromophore of VVD is flavin adenine dinucleotide (FAD) and VVD can rapidly form a homodimer from two monomers in response to blue light (Zoltowski *et al.*, 2007).

We previously designed PA-Gal4 transcriptional activators (PA-Gal4cc) based on the concept of split transcription factors, in which light-dependent interactions between another PA-protein interaction modules Cry2-CIB1 can reconstitute a split Gal4 DNA binding domain and p65 transcription activation domain (Yamada *et al.*, 2020). Although these PA-Gal4cc transcription factors also showed limited dark leakage and reliable light-dependent activity, due to the larger protein size of PA-Gal4cc, efficiency of plasmid transfection or viral transduction was not always high. Furthermore, in the case of PA-Gal4cc, two elements (i.e., Gal4 DBD-CIB1 and p65 AD-Cry2) were needed to introduced to cells that sometimes led to lower simultaneous transfection/transduction efficiency and limited expression levels. In contrast, the coding sequence size of eGAV is 1506 bp, and simple activation mechanism of eGAV allow it to achieve higher transfection efficiency and light-induced gene expression in cells.

We focused on the validation of eGAV-mediated gene expression control by one-photon activation of optogenetic switches. For the targeted manipulation of cells in tissues or organs, the two-photon activation of optogenetic switches could be promising (Kinjo *et al.*, 2019; Yao *et al.*, 2020; Guglielmi *et al.*, 2015; Kennedy *et al.*, 2010; Schindler *et al.*, 2015). Indeed, VVD or its derivative Magnet was applied to develop PA-site-specific DNA recombinases, such as Cre, Flp and Dre (Kawano *et al.*, 2016; Jung *et al.*, 2019; Morikawa *et al.*, 2020; Li *et al.*, 2020; Yao *et al.*, 2020). These PA-site-specific DNA recombinases were shown to be activated in cells by two-photon lasers in the tissues or organs, such as the brain. Therefore, optimization of two-photon activation of eGAV would enable precise temporal and spatial manipulation of targeted single cells or groups of cells in model organisms.

Since eGAV has faster temporal characteristics, switch-on kinetics, and significantly higher gene expression activity in response to a single light exposure than hGAVPO (Fig. 3), therefore eGAV can be applied to experiments requiring rapid activation of the gene of interest. For instance, we previously highlighted the functional contribution of dynamic gene expression changes in the bHLH-type transcription factor Ascl1 during the self-renewal and neuronal fate-determination of neural stem cells (Imayoshi *et al.*, 2013, Imayoshi and Kageyama, 2014a, 2014b). In these studies, we showed the unique utility of the PA-Gal4/UAS system to overcome technical limitations of conventional chemically regulated gene expression systems, such as limited reversibility and poor temporal control.

The observed on- and off-kinetics of light-induced gene expressions were sometime different in the adapted experimental model systems. For example, the faster on- and off- kinetics were observed in the mouse neural stem cells and progenitors compared with HEK293T cells. This might be due to the differences in gene delivery methods or animal species of cells used in the experiments. Also, the expression levels of PA-transcription factors and copy numbers of reporter sequences may affect the temporal characteristics of light-dependent gene expressions.

It has been shown that several point mutations of VVD or Magnet induce faster or slower photocycles (Zoltowski *et al.*, 2009; Nihongaki *et al.*, 2014; Benedetti *et al.*, 2020). Application of these effective mutations could lead to the development of eGAV variants having different switch-on/off kinetics, such as fast-cycling or step-function types. In our experience, optimization of PA-transcription factors always requires validations of many possible constructs (Yamada *et al.*, 2018, 2020), suggesting proper protein structures of reconstituted fused proteins are important for efficient light-dependent transcriptional activity. It is expected that attachment of large photoactivation modules induce conformational changes in the Gal4 DBD and transcription AD and inhibits gene expression activity. In addition, we have not examined linker sequences connecting Gal4 DBD, transcription AD and VVD in detail. Therefore, validation of the linker sequences in eGAV may further improve its functions.

In Fig. 2, we showed that blue light intensity-dependent increase of luciferase reporter activity in eGAV-expressing cells, but not in hGAVPO-expressing cells. As reported previously and also confirmed in this study, the leaky background activity of hGAVPO in dark is very high, suggesting significant proportions of inactive hGAVPOs in cells would partially make homodimer state even without blue light illumination. Therefore, even short-time or weak blue-light irradiation can fully activate almost all proportions of kind of primed hGAVPO existing in cells. This extreme sensitivity of hGAVPO is sometimes advantageous in the experiments where the delivery of blue light is technically limited, such as deep inside the large tissues. Also, leak transcription activity of hGAVPO in dark may not cause significant problems in the experiments the basal levels of downstream gene expression is rather anticipated. These findings suggest that hGAVPO is more suitable for experiments in which stable cells expressing this factor at not too-high levels can be prospectively screened and identified. In contrast, due to the lower dark background activity of eGAV, this is more advantageous for transient transfection experiments where the rigorous control of PA-transcription factor expression levels is more difficult, and transfection efficiencies are more variable between cells.

In summary, eGAV can be introduced into cells by various methods including lipofection, electroporation, hydrodynamic-based transfection and lentivirus vectors. We demonstrated reliable blue light-controllable gene expression in *in vitro* and *in vivo* models. Compared to the widely used PA-Gal4 transcription factor hGAVPO, the dark background activity of eGAV was significantly lower and the maximum light-induced gene expression level was higher, thus improving the reliability of light-induced gene expression controls. Therefore, we believe that eGAV will be a valuable tool for the systematic analysis of dynamic changes in cellular gene expression during morphological, functional, and pathological changes in multicellular systems.

## Author Contributions

S.C.N. and I.I. conceived the project and designed the experiments. S.C.N. performed most of the experiments. T.D.F. and S.C.N. performed the eGAV-eMag variant evaluation experiments. A.T.G. and S.C.N. performed the *in ovo* electroporation experiments. Y.S.III and S.C.N. conducted data analysis. M.Y., R.K. and I.I. supervised the experiments to evaluate the optogenetic tools. S.C.N., A.T.G. and I.I. wrote the manuscript with input from all of the other authors.
