# Supplementary material for: Enhancement of Vivid-based photo-activatable Gal4 transcription factor in mammalian cells
Source: Cell Struct Funct. 2022 Dec 16;48(1):31–47. doi: 10.1247/csf.22074 (PMC10721950; doi:10.1247/csf.22074)
Supplement: Supplementary file 1 — Supplementary Materials [file csf_48_22074_1.pdf]

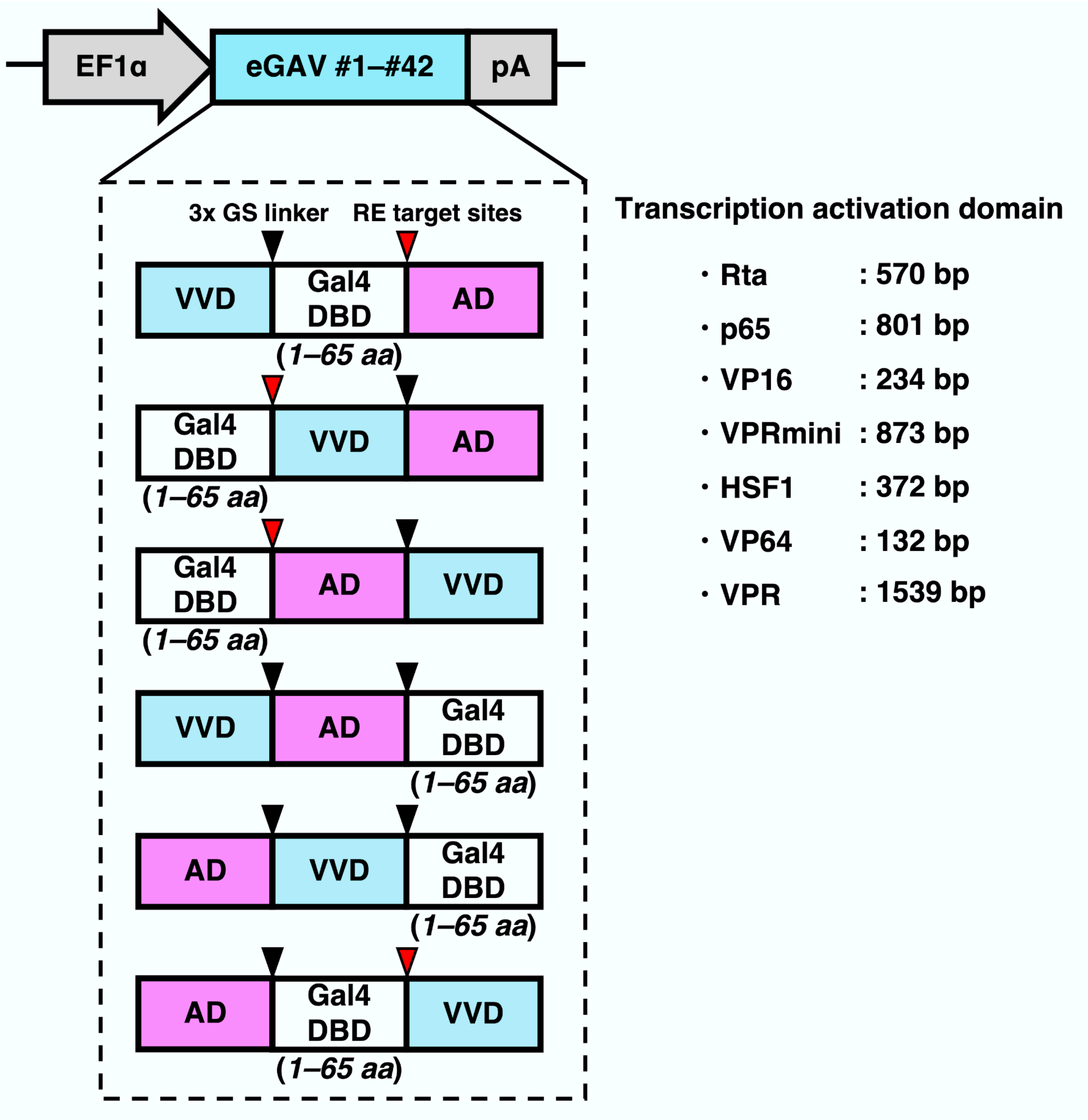

Fig. S1. Schematic representation of eGAV candidate constructs, related to Fig. 1. Cyan boxes indicate blue light-dependent homodimer formation molecule Vivid (VVD), and magenta boxes indicate transcription activation domain (AD). The residues 1–65 of Gal4 DNA-binding domain (DBD) was used. The three tandem flexible Glycine–Serine (3x GS) linker, indicated by black arrowheads, or restriction enzyme (RE) target sites, indicated by red arrowheads, were inserted between each protein domain. Possible tested six configurations of the three functional domains and seven adapted transcription AD were displayed. In total, 42 constructs in Table S1 were subjected to functional screenings in HEK293T cells.

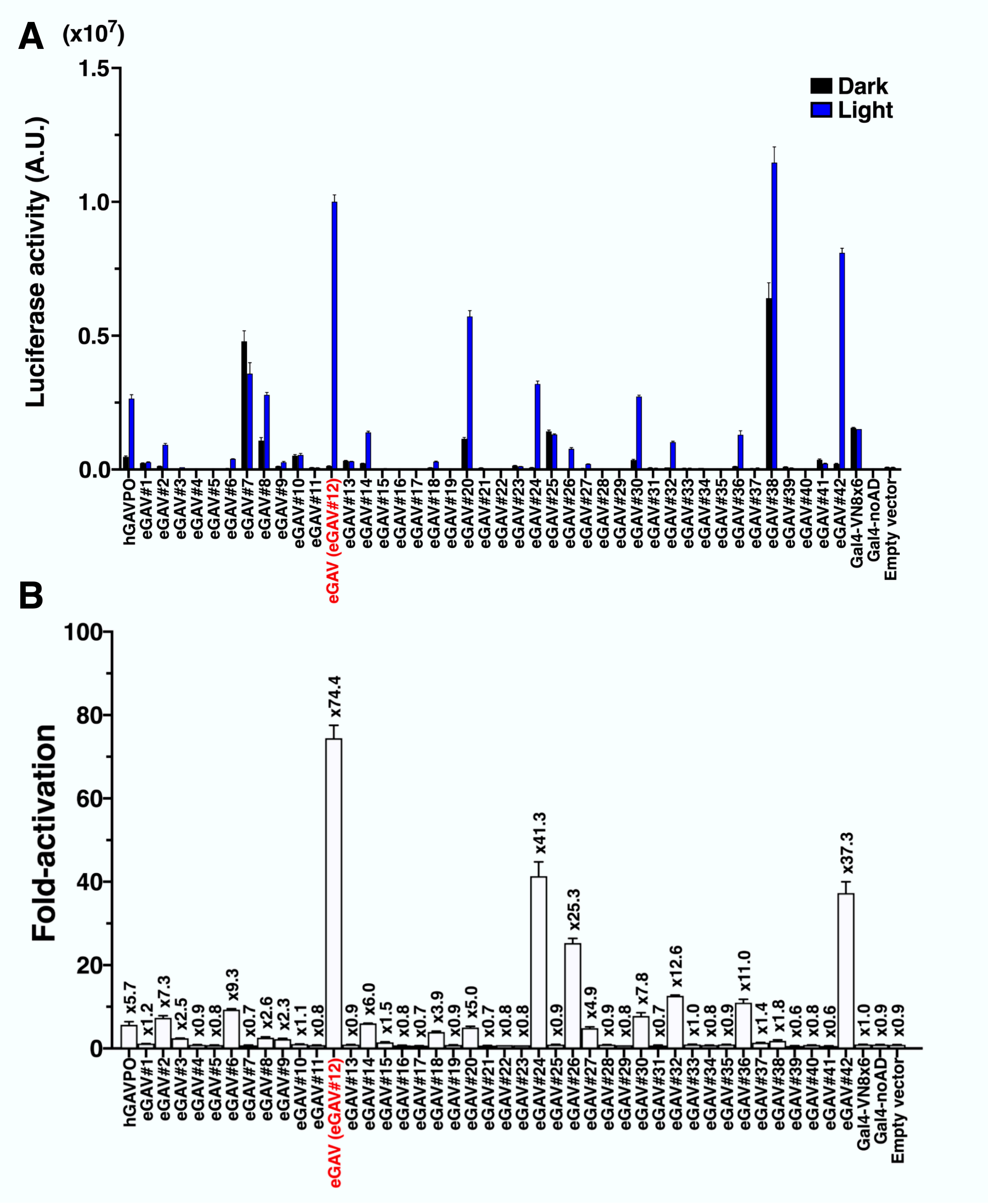

Fig. S2. Functional screening of eGAV candidate constructs, related to Fig. 1. eGAV candidate constructs with the 5x UAS-Ub-NLS-Luc2-Hes1 3′ UTR reporter were transfected into HEK293T cells, and their light-dependent transcriptional activities were assayed. As negative internal controls, samples transfected with the light non-sensitive Gal4 transcription factor Gal4-VN8x6 (Salghetti et al., 2000), Gal4 DBD without any transcription AD, and empty expression vector were used. The data represent means ± standard deviation (SD) (n = 3) from one experiment, and experiments were repeated three times with similar results. See also Table S2.

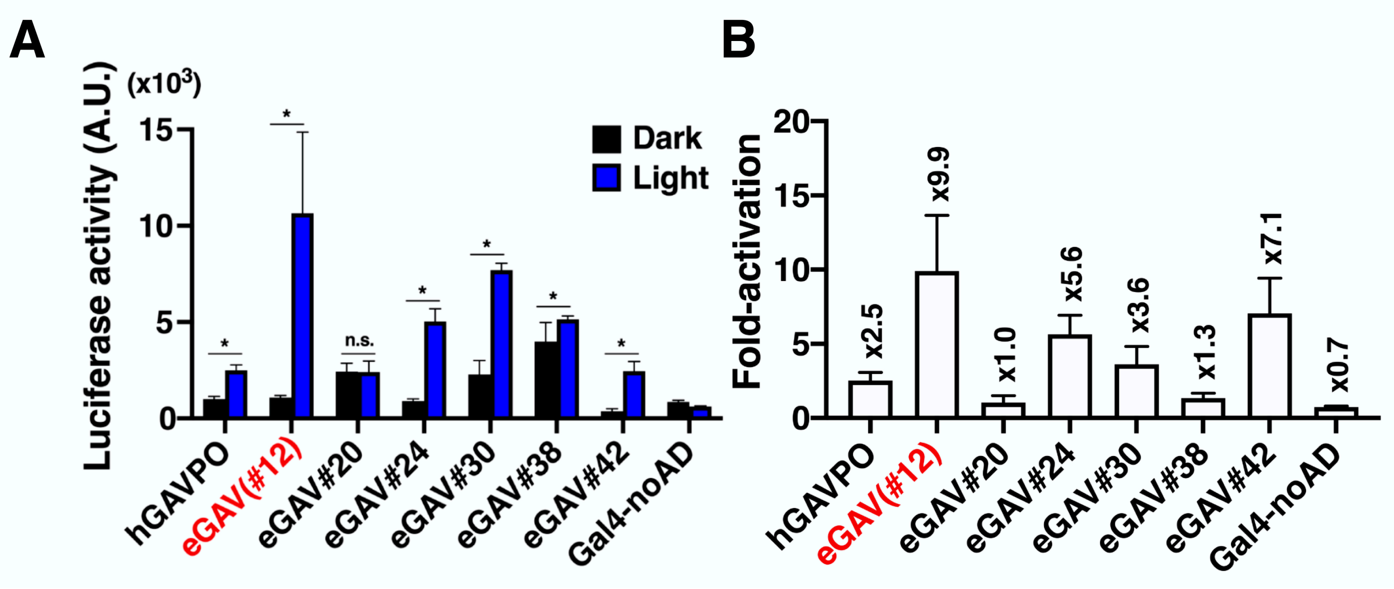

Fig. S3. Validation of the selected eGAV candidate constructs in NIH3T3 cells. (**A**) Validation of light-dependent regulation of the eGAV constructs in transiently-transfected NIH3T3 cells. Six candidate constructs which showed prominent light-induced expressions in HEK293T cells were selected. Similar to the results of experiments using HEK293T cells, the eGAV#12 showed the highest fold induction of the luciferase reporter among the eGAV candidates. Student *t*-test was used to compare the reporter activity of hGAVPO or each eGAV construct in dark and light conditions (*p<0.05). (**B**) Fold-increase of luciferase activity (Light/Dark). The data represent means ± SD (n = 3) from one experiment, and experiments were repeated three times with similar results.

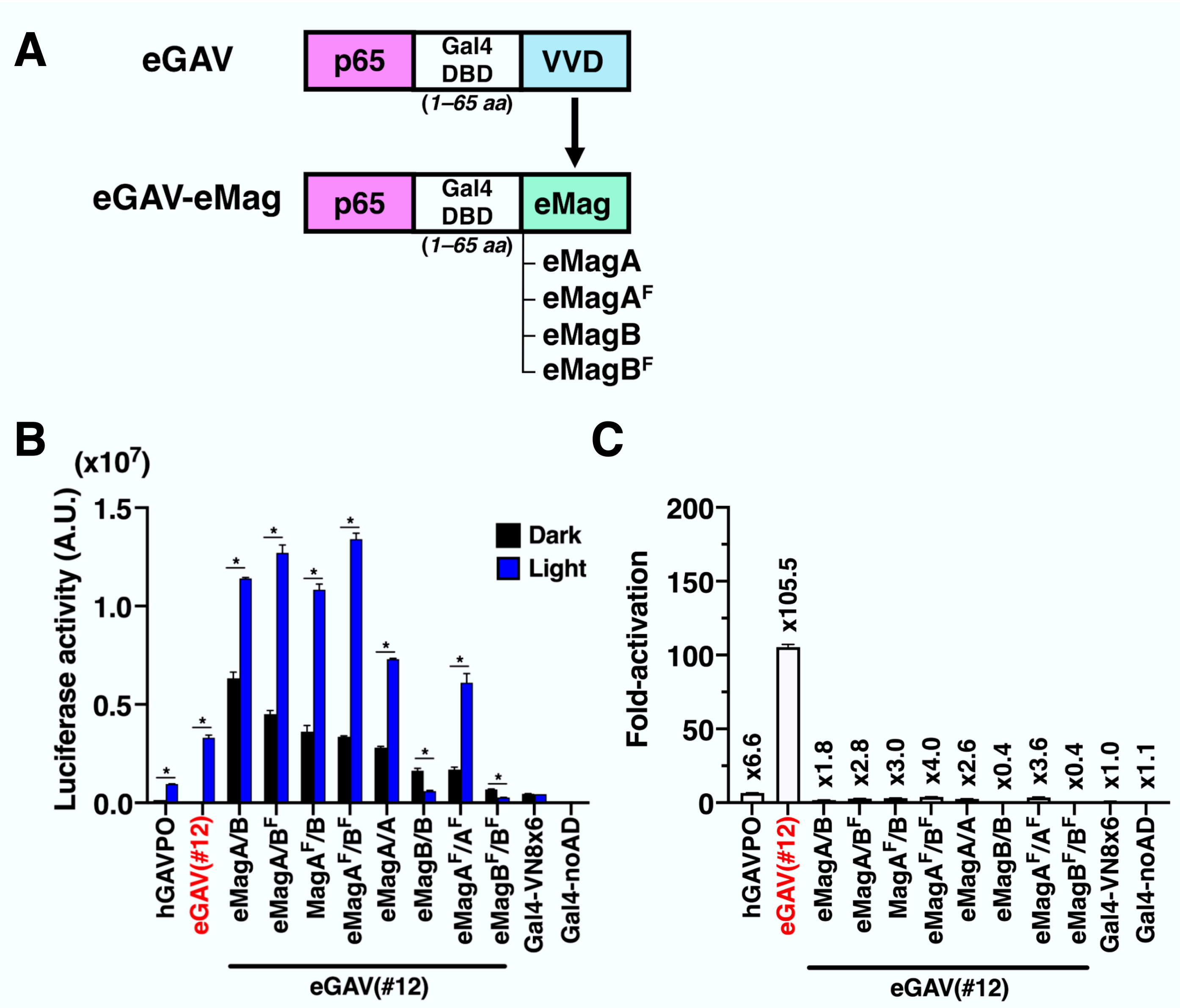

Fig. S4. The Magnet variants does not substitute for VVD in the eGAV-mediated light-dependent transcription. (**A**) The VVD sequence of eGAV#12 construct was changed to eMagnet (eMag), which is enhanced thermostable variants of Magnet. However, any pairs of eGAV-Mag showed the extremely high levels of background even in the dark condition. This led to the poor fold activation ratio of eGAV-eMag constructs. Student *t*-test was used to compare the reporter activity of each PA-Gal4 construct in dark and light conditions (*p<0.05). (**B**) The data represent means ± SD (n = 3) from one experiment, and experiments were repeated three times with similar results.

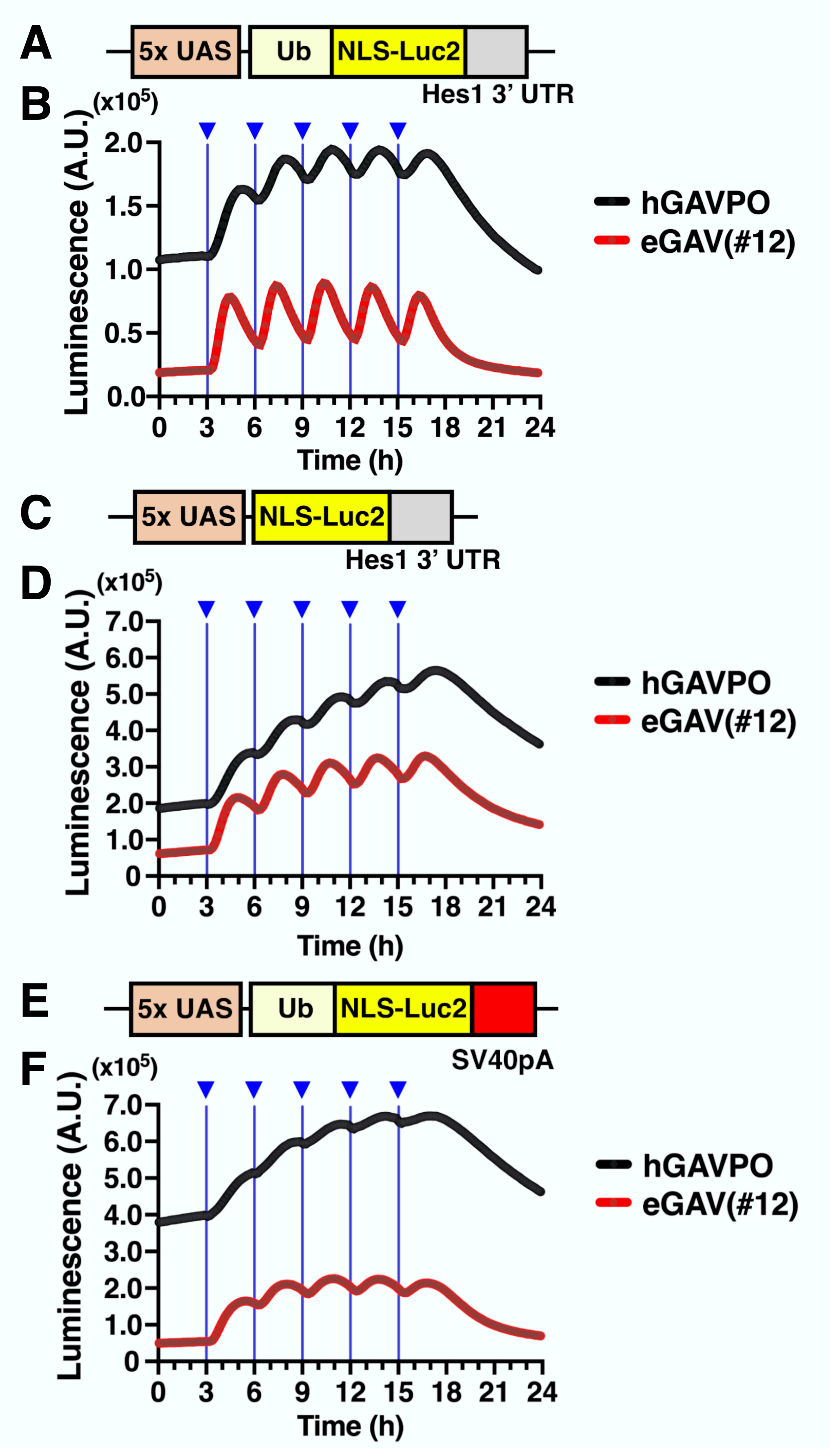

Fig. S5. Periodic activation of eGAV transcriptional activators. (**A-F**) HEK293T cells were transiently transfected with eGAV and repeatedly exposed to blue light pulses at 3 h intervals. 5x UAS-Ub-NLS-Luc2-Hes1 3′ UTR reporter (**A, B**), 5x UAS-NLS-Luc2-Hes1 3′ UTR reporter (**C, D**) or 5x UAS-Ub-NLS-Luc2-SV40pA reporter (**E, F**) were used. The timing of blue light exposure is indicated by vertical blue lines. The first blue light illumination was initiated 36 h after the transfection. Experiments were repeated at least three times with similar results (*n* = 6). The eGAV-transfected cells showed lower basal luciferase expressions and clear periodic reporter activity changes compared with hGAVPO-transfected cell.

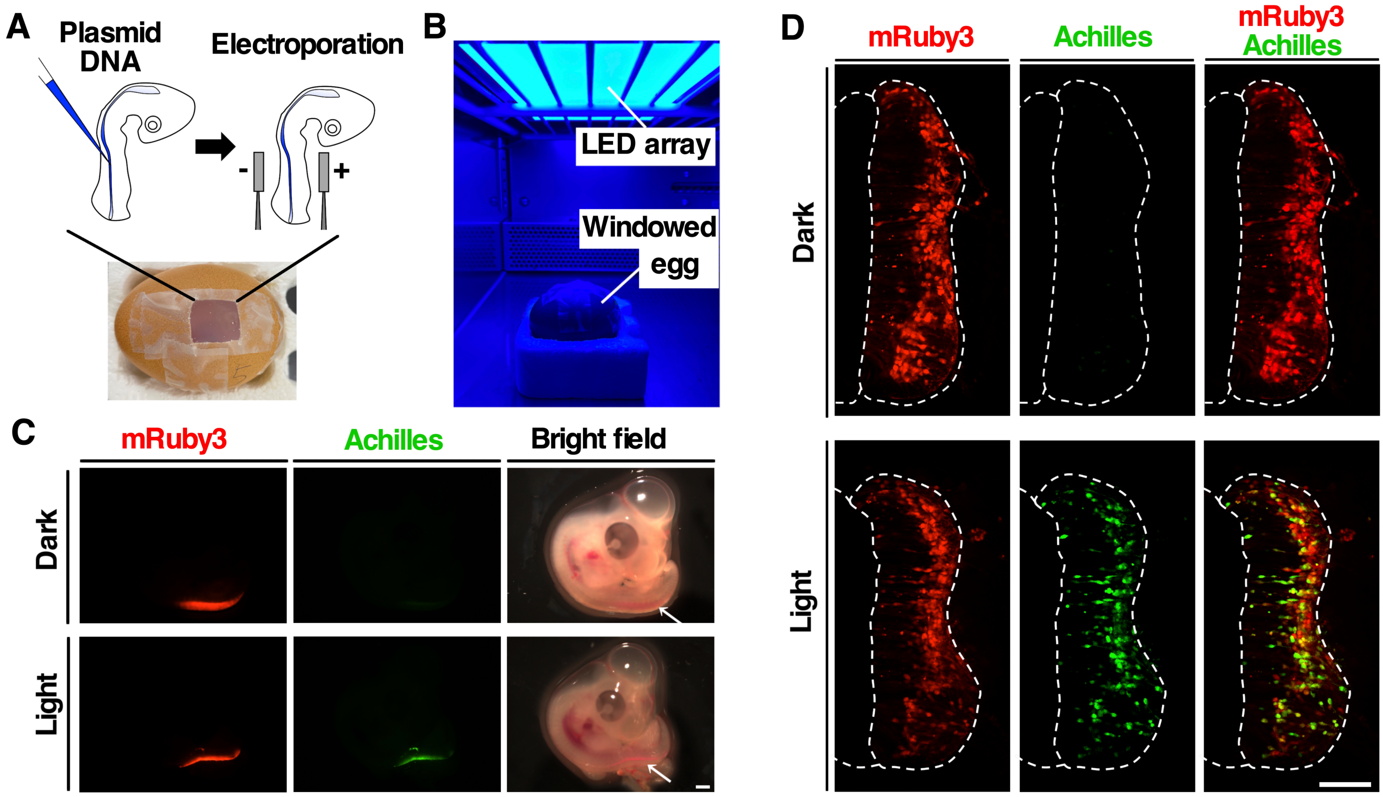

Fig. S6. Blue-light dependent gene expression activity of eGAV in the developing chicken spinal cord. (**A**) The plasmid mixture of eGAV expression vector, 5xUAS-Achilles-NLS-PEST-Hes1 3' UTR reporter and mRuby3 transfection marker expression vector was injected into the central canal of the spinal cord of HH17-18 chicken embryo *in ovo*, followed by unilateral electroporation. (**B**) Embryos were irradiated with blue light through the egg shell window using an LED array. (**C**) Under the blue light condition, Achilles reporter expression was observed in the transfected region of spinal cord (white arrow), but not in the dark condition. (**D**) Transverse sections of the electroporated spinal cords, displaying light-dependent Achille expressions in the mRuby3-positive transfected cells. Scale bars, 1 mm for (**C**) and 100 µm for (**D**).

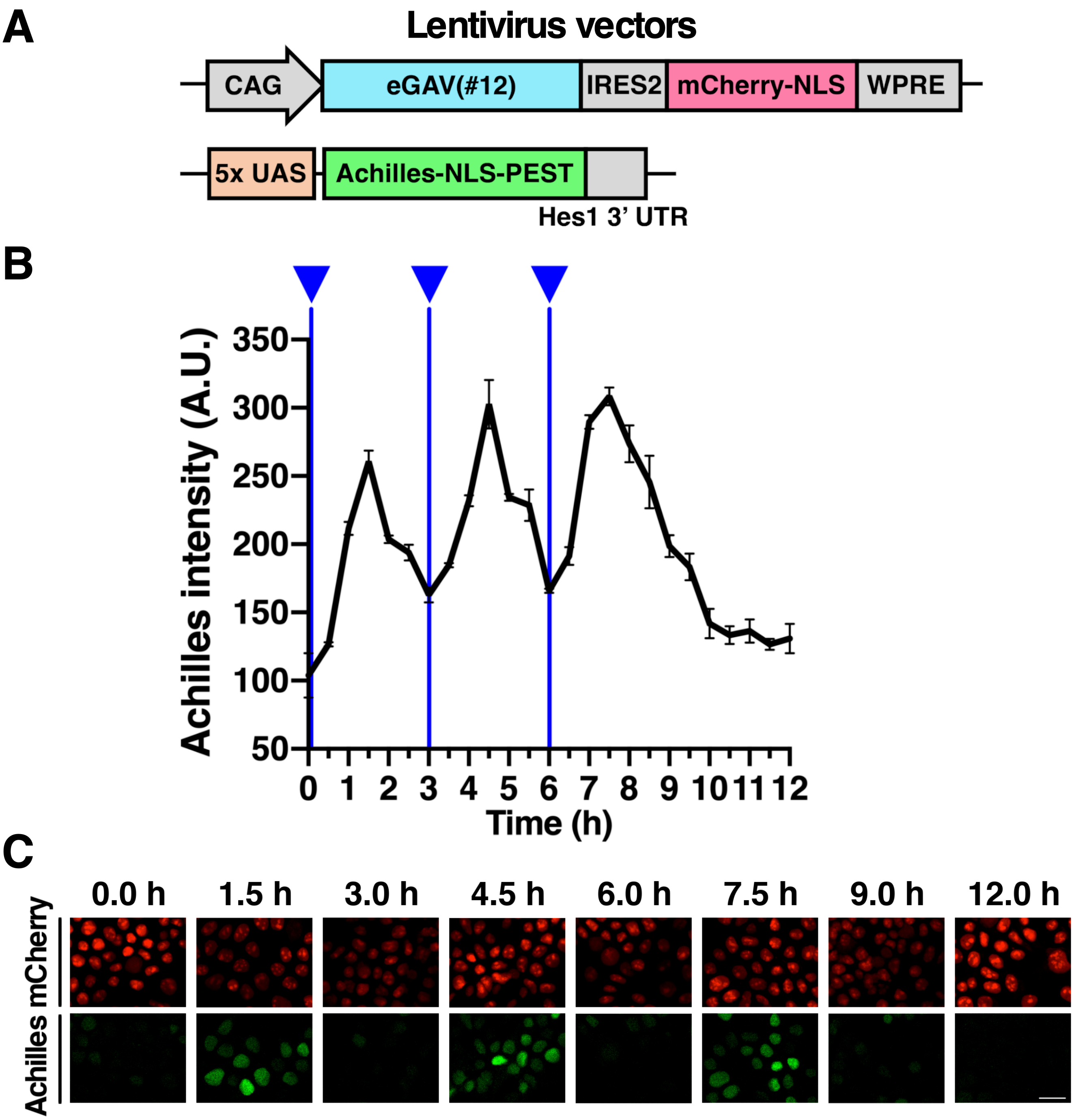

Fig. S7. Evaluation of periodic activation of eGAV using the 5x UAS-Achilles-NLS-PEST-Hes1 3' UTR reporter in HEK293T cells. (**A**) The eGAV-expressing stable HEK293T cells were generated by infection with lentivirus vectors, CAG-eGAV-IRES2-mCherry-NLS-WPRE and 5x UAS-Achilles-NLS-PEST-Hes1 3' UTR reporter. (**B**) The reporter activity changes by blue-light irradiation were quantified at each time point with the flow cytometer. The timing of blue light exposure is indicated by vertical blue lines. The data represent means ± SD (n = 3). (**C**) Representative fluorescence images of HEK293T cells at each time point were displayed. Scale bar, 20 µm.

Table S1. THE CONSTRUCT SUMMARY OF eGAV CANDIDATES

Construct ID	Domain#1	Linker#1	Domain#2	Linker#2	Domain#3
eGAV#1	VVD	3x GS linker-XbaI	Gal4 DBD	SpeI-MluI	Rta
eGAV#2	Gal4 DBD	SpeI-MluI	VVD	3x GS linker-XbaI	Rta
eGAV#3	Gal4 DBD	SpeI-MluI	Rta	3x GS linker-XbaI	VVD
eGAV#4	VVD	3x GS linker-XbaI	Rta	3x GS linker-SpeI-MluI	Gal4 DBD
eGAV#5	Rta	3x GS linker-XbaI	VVD	3x GS linker-SpeI-MluI	Gal4 DBD
eGAV#6	Rta	3x GS linker-SpeI-MluI	Gal4 DBD	XbaI	VVD
eGAV#7	VVD	3x GS linker-XbaI	Gal4 DBD	SpeI-MluI	p65
eGAV#8	Gal4 DBD	SpeI-MluI	VVD	3x GS linker-XbaI	p65
eGAV#9	Gal4 DBD	SpeI-MluI	p65	3x GS linker-XbaI	VVD
eGAV#10	VVD	3x GS linker-XbaI	p65	3x GS linker-SpeI-MluI	Gal4 DBD
eGAV#11	p65	3x GS linker-XbaI	VVD	3x GS linker-SpeI-MluI	Gal4 DBD
eGAV (eGAV#12)	p65	3x GS linker-SpeI-MluI	Gal4 DBD	XbaI	VVD
eGAV#13	VVD	3x GS linker-XbaI	Gal4 DBD	SpeI-MluI	VP16
eGAV#14	Gal4 DBD	SpeI-MluI	VVD	3x GS linker-XbaI	VP16
eGAV#15	Gal4 DBD	SpeI-MluI	VP16	3x GS linker-XbaI	VVD
eGAV#16	VVD	3x GS linker-XbaI	VP16	3x GS linker-SpeI-MluI	Gal4 DBD
eGAV#17	VP16	3x GS linker-XbaI	VVD	3x GS linker-SpeI-MluI	Gal4 DBD
eGAV#18	VP16	3x GS linker-SpeI-MluI	Gal4 DBD	XbaI	VVD
eGAV#19	VVD	3x GS linker-XbaI	Gal4 DBD	SpeI-MluI	VPRmini
eGAV#20	Gal4 DBD	SpeI-MluI	VVD	3x GS linker-XbaI	VPRmini
eGAV#21	Gal4 DBD	SpeI-MluI	VPRmini	3x GS linker-XbaI	VVD
eGAV#22	VVD	3x GS linker-XbaI	VPRmini	3x GS linker-SpeI-MluI	Gal4 DBD
eGAV#23	VPRmini	3x GS linker-XbaI	VVD	3x GS linker-SpeI-MluI	Gal4 DBD
eGAV#24	VPRmini	3x GS linker-SpeI-MluI	Gal4 DBD	XbaI	VVD
eGAV#25	VVD	3x GS linker-XbaI	Gal4 DBD	SpeI-MluI	HSF1
eGAV#26	Gal4 DBD	SpeI-MluI	VVD	3x GS linker-XbaI	HSF1
eGAV#27	Gal4 DBD	SpeI-MluI	HSF1	3x GS linker-XbaI	VVD
eGAV#28	VVD	3x GS linker-XbaI	HSF1	3x GS linker-SpeI-MluI	Gal4 DBD
eGAV#29	HSF1	3x GS linker-XbaI	VVD	3x GS linker-SpeI-MluI	Gal4 DBD
eGAV#30	HSF1	3x GS linker-SpeI-MluI	Gal4 DBD	XbaI	VVD
eGAV#31	VVD	3x GS linker-XbaI	Gal4 DBD	SpeI-MluI	VP64o
eGAV#32	Gal4 DBD	SpeI-MluI	VVD	3x GS linker-XbaI	VP64o
eGAV#33	Gal4 DBD	SpeI-MluI	VP64o	3x GS linker-XbaI	VVD
eGAV#34	VVD	3x GS linker-XbaI	VP64o	3x GS linker-SpeI-MluI	Gal4 DBD
eGAV#35	VP64	3x GS linker-XbaI	VVD	3x GS linker-SpeI-MluI	Gal4 DBD
eGAV#36	VP64o	3x GS linker-SpeI-MluI	Gal4 DBD	XbaI	VVD
eGAV#37	VVD-GS	XbaI	Gal4 DBD	SpeI-MluI	VPR
eGAV#38	Gal4 DBD	SpeI-MluI	VVD	3x GS linker-XbaI	VPR
eGAV#39	Gal4 DBD	SpeI-MluI	VPR	3x GS linker-XbaI	VVD
eGAV#40	VVD	3x GS linker-XbaI	VPR	3x GS linker-SpeI-MluI	Gal4 DBD
eGAV#41	VPR	3x GS linker-XbaI	VVD	3x GS linker-SpeI-MluI	Gal4 DBD
eGAV#42	VPR	3x GS linker-SpeI-MluI	Gal4 DBD	XbaI	VVD

Table S1. The construct summary of eGAV candidates, related to Fig. 1. Summary of the configuration, applied transcription AD, and the inserted restriction enzyme (RE)-target sites and 3x GS linkers of individual eGAV candidate constructs.

Table S2. SUMMARY FOR VALIDATION OF eGAV CONSTRUCTS IN TRANSIENTLY-TRANSFECTED HEK293T CELLS

Construct ID	Light/Dark ratio		Dark		Light		Unpaired *t*-test *p* value
	Mean	SD	Mean	SD	Mean	SD	
hGAVPO	5.7	0.8	470000	35679	2650000	147309	<0.0001
eGAV#1	1.2	0.0	236667	18037	272667	15948	0.0607
eGAV#2	7.3	0.5	126333	3786	923667	50362	<0.0001
eGAV#3	2.5	0.0	35133	493	86433	666	<0.0001
eGAV#4	0.9	0.0	32933	1474	29333	723	0.0192
eGAV#5	0.8	0.0	23233	635	19133	321	0.0006
eGAV#6	9.3	0.2	42467	1415	395667	6028	<0.0001
eGAV#7	0.7	0.1	4786667	398790	3583333	411015	0.0220
eGAV#8	2.6	0.2	1080667	114548	2790000	90000	<0.0001
eGAV#9	2.3	0.2	121333	8386	273333	28746	0.0009
eGAV#10	1.1	0.0	517000	40447	545667	56048	0.5123
eGAV#11	0.8	0.0	79967	2558	66567	3907	0.0077
eGAV (eGAV#12)	74.4	3.1	134667	9074	10006667	254231	<0.0001
eGAV#13	0.9	0.0	332667	9713	305333	5033	0.0124
eGAV#14	6.0	0.0	230333	6807	1386667	46188	<0.0001
eGAV#15	1.5	0.1	25000	600	36633	1914	0.0006
eGAV#16	0.8	0.0	21733	1301	16333	451	0.0025
eGAV#17	0.7	0.0	30833	1102	20333	1419	0.0005
eGAV#18	3.9	0.2	73800	2685	291333	25423	0.0001
eGAV#19	0.9	0.0	13067	808	11200	1058	0.0721
eGAV#20	5.0	0.3	1146667	51316	5720000	216564	<0.0001
eGAV#21	0.7	0.0	67033	1332	45167	1102	<0.0001
eGAV#22	0.8	0.0	8210	113	6667	178	0.0002
eGAV#23	0.8	0.0	148667	4163	116333	2082	0.0003
eGAV#24	41.3	3.4	77533	4020	3196667	106927	<0.0001
eGAV#25	0.9	0.0	1420000	52915	1306667	28868	0.0312
eGAV#26	25.3	1.1	30533	611	772667	48418	<0.0001
eGAV#27	4.9	0.3	42700	3124	206667	3215	<0.0001
eGAV#28	0.9	0.0	11267	503	10210	543	0.0689
eGAV#29	0.8	0.0	38767	1290	30067	1266	0.0011
eGAV#30	7.8	0.8	352333	30436	2730000	51962	<0.0001
eGAV#31	0.7	0.1	71700	5057	51600	2972	0.0040
eGAV#32	12.6	0.2	80833	2178	1022333	36556	<0.0001
eGAV#33	1.0	0.1	52133	1002	50567	2098	0.3080
eGAV#34	0.8	0.0	60500	1136	48600	1044	0.0002
eGAV#35	0.9	0.0	31500	1114	29533	208	0.0397
eGAV#36	11.0	0.8	118000	5196	1296667	153080	0.0002
eGAV#37	1.4	0.1	41433	2346	57000	5839	0.0128
eGAV#38	1.8	0.2	6400000	575587	11466667	585947	0.0004
eGAV#39	0.6	0.1	100967	5348	59900	4480	0.0005
eGAV#40	0.8	0.0	23267	757	19567	839	0.0048
eGAV#41	0.6	0.0	364000	28213	220333	18009	0.0017
eGAV#42	37.3	2.7	217667	13650	8093333	171561	<0.0001
Gal4-VN8x6	1.0	0.0	1560000	17321	1510000	0	0.0075
Gal4-noAD	0.9	0.0	23100	361	21333	208	0.0018
Empty vector	0.9	0.0	90667	2250	80100	5742	0.0412

Table S2. Summary for validation of eGAV constructs in transiently-transfected HEK293T cells, related to Fig. 1. eGAV candidate constructs with the 5x UAS-Ub-NLS-Luc2-Hes1 3′ UTR reporter were transfected into HEK293T cells, and their light-dependent transcriptional activities were assayed (**Fig. S2**). The data represent mean values ± SD ($n = 3$). Student *t*-test was used to compare the reporter activity of each construct in dark and light conditions.
